# Fat and fatty acids – a scoping review for Nordic Nutrition Recommendations 2023

**DOI:** 10.29219/fnr.v68.9980

**Published:** 2024-01-12

**Authors:** Kjetil Retterstøl, Fredrik Rosqvist

**Affiliations:** 1 Department of Nutrition, Institute of Basic Medical Sciences, University of Oslo, Oslo, Norway; 2 Department of Public Health and Caring Sciences, Clinical Nutrition and Metabolism, Uppsala University, Uppsala, Sweden

**Keywords:** fatty acids, lipids, cardiovascular disease, type 2 diabetes, recommendations

## Abstract

Two *de novo* NNR2022 systematic reviews (SRs) as well as 21 qualified SRs (qSRs) were available. A literature search yielded an additional ~70 SRs, meta-analyses and biomarker papers. Diets lower in total fat are associated with reductions in body weight and blood pressure compared with diets higher in total fat in adults. Partial replacement of saturated fatty acid (SFA) with n-6 polyunsaturated fatty acid (PUFA) improves blood lipid profile, decreases the risk of cardiovascular disease (CVD), improves glucose-insulin homeostasis and may decrease the risk of total mortality. Long-chain n-3 PUFAs (eicosapentaenoic acid [EPA] and docosahexaenoic acid [DHA]) decrease triglycerides and are associated with lower risk of CVD. Dietary PUFAs, both n-3 and n-6, may be associated with reduced risk of type 2 diabetes (T2D). There is inconclusive evidence to suggest that the type of dietary fat is associated with blood pressure, risk of hypertension or musculoskeletal health. Higher intake of total PUFA is associated with lower mortality from any cancer. Long-chain n-3 PUFA is associated with reduced risk of breast cancer, whereas biomarker levels of n-6 PUFA are associated with lower risk of any cancer. Intake of long-chain n-3 PUFA during pregnancy increases length of gestation and child birth weight and reduces the risk of preterm delivery, but there is inconclusive evidence to suggest that it may influence child neurodevelopment, growth or development of allergic disease. In studies with higher versus lower dietary cholesterol intake levels, total blood cholesterol increased or were unaffected by the dietary cholesterol, resulting in inconclusive results. Trans fatty acid (TFA), regardless of source, impairs blood lipid profile compared to unsaturated fat. In observational studies, TFA is positively associated with CVD and total mortality but whether associations differ by source is inconclusive. Ruminant TFA, as well as biomarker levels of odd-chain fatty acids, might be associated with lower risk of T2D.

## Popular scientific summary

- The intake of total fat in the Nordic and Baltic countries varies from 34 to 43.7% of total energy in men and from 34 to 42.1% in women.
- Replacing saturated fatty acids with n-6 polyunsaturated fatty acids improves blood lipids and lowers cardiovascular disease risk.
- Long-chain n-3 polyunsaturated fatty acids decrease triglycerides and are associated with lower risk of cardiovascular diseases.
- Higher biomarker levels of polyunsaturated fats are associated with lower risk of type 2 diabetes.

Fat provides the body with energy in a concentrated form. In addition to energy, dietary fats provide essential fatty acids and fat-soluble vitamins. Lipids, mainly phospholipids and cholesterol, are included in cell membranes, and triglycerides (three fatty acids esterified to glycerol) are stored in adipose tissue as energy reserves. Certain fatty acids serve as a source of eicosanoids and other signaling molecules. Certain fatty acids may also exert signaling functions by themselves. Thus, the composition of dietary fat influences the biochemical milieu in the body. In food items, fats are usually in the form of triglycerides, which consist of three fatty acids bound to one molecule of glycerol. The physiological effects of different fatty acids within triglycerides depend on the length of the carbon chain, the degree of saturation, the number, position and structure of the double bonds, and, to some extent, their position in the triglyceride molecule. The unsaturated fatty acids are characterized by the number of double bonds in the molecule: monounsaturated fatty acids (MUFAs) have only one double bond whereas polyunsaturated fatty acids (PUFAs) have 2–6 double bonds. Saturated fatty acids (SFAs) have no double bonds. Trans fatty acids (TFAs) are unsaturated fatty acids characterized by one or more double bonds in transconfiguration, chemically formed by partial hydrogenation and deodorization of vegetable and fish oils (industrial TFA). They are also formed by natural biohydrogenation of fatty acids in the rumen of cattle, sheep, and goats (ruminant TFA) and, therefore, are present in milk and meat. The aim of this scoping review is to describe the totality of evidence for the role of fat and fatty acids for health-related outcomes as a basis for setting and updating DRVs (Box 1).

*Box 1.* General information regarding the Nordic Nutrition Recommendations 2023 (NNR2023) project

- This paper is one of many scoping reviews commissioned as part of the Nordic Nutrition Recommendations 2023 (NNR2023) project (1).
- The papers are included in the extended NNR2023 report but, for transparency, these scoping reviews are also published in Food & Nutrition Research.
- The scoping reviews have been peer reviewed by independent experts in the research field according to the standard procedures of the journal.
- The scoping reviews have also been subjected to public consultations (see report to be published by the NNR2023 project).
- The NNR2023 committee has served as the editorial board.
- While these papers are a main fundament, the NNR2023 committee has the sole responsibility for setting dietary reference values in the NNR2023 project.

## Methods

This review follows the protocol developed within the NNR2023 project (1). The sources of evidence used in this scoping review follow the eligibility criteria described in the paper ‘The Nordic Nutrition Recommendations 2022 – Principles and methodologies’ (2). Systematic review (SRs) articles that do not fulfill the criteria for qualified systematic reviews (qSRs) according to the NNR2023 project are defined as non-qSR articles. This also applies to studies of high quality in accordance with the AMSTAR 2 evaluation tool. Two *de novo* NNR2023 SRs (‘Fat quality and mental health’ and ‘Intake of n-3 PUFA from supplements during pregnancy and asthma and allergies in the offspring’) as well as 21 previously published qSRs are included as a source of evidence in this scoping review. These 21 qSRs are presented in Table 1. Furthermore, a literature search for SRs and meta-analyses was conducted in PubMed on 24 August 2021 (updated on 04 February 2022) using the following search string: ((“fat”[Title] OR “fatty acids”[Title]) AND (“diet”[MeSH Terms] OR “diet”[All Fields] OR (“diet”[MeSH Terms] OR “diet”[All Fields] OR “dietary”[All Fields] OR “dietaries”[All Fields]) OR (“food”[MeSH Terms] OR “food”[All Fields]) OR (“nutritions”[All Fields] OR “nutritional status”[MeSH Terms] OR (“nutritional”[All Fields] AND “status”[All Fields]) OR “nutritional status”[All Fields] OR “nutrition”[All Fields] OR “nutritional sciences”[MeSH Terms] OR (“nutritional”[All Fields] AND “sciences”[All Fields]) OR “nutritional sciences”[All Fields] OR “nutritional”[All Fields] OR “nutritionals”[All Fields] OR “nutritions”[All Fields] OR “nutritive”[All Fields]) OR (“nutrition s”[All Fields] OR “nutritional status”[MeSH Terms] OR (“nutritional”[All Fields] AND “status”[All Fields]) OR “nutritional status”[All Fields] OR “nutrition”[All Fields] OR “nutritional sciences”[MeSH Terms] OR (“nutritional”[All Fields] AND “sciences”[All Fields]) OR “nutritional sciences”[All Fields] OR “nutritional”[All Fields] OR “nutritionals”[All Fields] OR “nutritions”[All Fields] OR “nutritive”[All Fields]))) AND ((meta-analysis[Filter] OR systematicreview[Filter]) AND (humans[Filter]) AND (2011:2021[pdat])). The search resulted in 417 hits, of which 148 were selected for further assessment. Study selection was done in duplicate and consensus was reached through discussion. The search was repeated without filtering for ‘Humans,’ which resulted in an additional six papers of relevance. A few SRs and biomarker papers, not picked up by the search, were also considered. The quality of the included studies (Table 2) was evaluated using the tool AMSTAR2 (3). For certain topics (e.g. cardiovascular disease [CVD]), many of these search hits were already covered by one or several of the qSRs and therefore not further considered. The reasons for excluding specific studies are provided in Table 3. The scientific literature on fat and fatty acids is extensive, but the topics discussed in this scoping review are to some degree defined by the topics covered by the qSRs. In case of multiple and overlapping qSRs, we chose the most recent and most updated qSRs over the older, or used both if they were complementary. The same principle was applied for non-qSRs.

**Table 1:** Systematic reviews

First author, year (reference)	Exposure	Main findings/conclusion
Abdelhamid, 2018 (28)	n-3 PUFA	EPA and DHA has little or no effect on CVD.
Abdelhamid, 2020 (29)	n-3 PUFA	EPA, DHA, and ALA may be slightly protective of CVD. Little or no effect on adiposity, lipids, and blood pressure, except increasing long-chain-3 PUFA reduced triglycerides by ˜15%. Qualified systematic review.
Abdelhamid, 2019 (102)	n-3, n-6, and total PUFA	Little or no effect on bone health, muscle mass, and functional status. Qualified systematic review.
Ajabnoor, 2021 (107)	n-3 and n-6 PUFA	PUFA has little or no effect on prevention or treatment of inflammatory bowel disease. Qualified systematic review.
Al-Khudairy, 2015 (25)	n-6 PUFA	No effects on blood lipids or blood pressure. No included trials reported CVD events.
Balk, 2016 (30)	n-3 PUFA	Long-chain n-3 PUFA or ALA did not clearly reduce ischemic stroke, hemorrhagic stroke, angina pectoris, MI, CVD, CHD, congestive heart failure. Qualified systematic review.
Brainard, 2020 (104)	n-3, n-6, and total PUFA	Long-chain n-3 PUFA probably has little or no effect on new neurocognitive outcomes or cognitive impairment. Qualified systematic review.
Brouwer, 2016 (53)	Trans fat (TFA)	Replacement of TFA by MUFA, PUFA, or carbohydrate improves levels of HDL and LDL, with PUFA showing the strongest effect. Replacement of TFA by SFA increased LDL and reduced TC/HDL and LDL/HDL. Qualified systematic review.
Brown, 2019 (59)	n-3, n-6, and total PUFA	Increasing n-3, n-6, or total PUFA has little or no effect on prevention and treatment of type 2 diabetes. Qualified systematic review.
Deane, 2021 (106)	n-3 PUFA supplementation	Supplementation with long-chain n-3 PUFA probably has little or no effect in preventing depression. Qualified systematic review.
de Souza, 2015 (50)	SFA and TFA	TFAs are associated with all-cause mortality, total CHD, and CHD mortality. SFAs are not associated with all-cause mortality, CVD, CHD, ischemic stroke, or type 2 diabetes, but the evidence is heterogeneous with methodological limitations. Qualified systematic review.
Delgado-Noguera, 2015 (128)	Long-chain PUFA in mother’s diet during the pregnancy and 4 months after birth	Inconclusive evidence to support or refute the practice of giving long-chain PUFA supplementation to breastfeeding mothers in order to improve neurodevelopment.
DGAC, 2020 (27)	Types of fat	Replacing SFA by UFA reduces LDL. Replacing SFA by carbohydrates reduces LDL but increases TG and reduces HDL. Replacing SFA with PUFA reduces CHD events and CVD mortality. Intake of LCn-3 from food sources is associated with lower risk of CVD. Qualified systematic review.
DGAC, 2020 (129)	n-3 PUFA supplementation	Limited evidence suggests that n-3 PUFA supplementation during pregnancy may result in favorable cognitive development in the child. Insufficient evidence on motor and visual development, academic performance, risk of ADD/ADHD/autism in the child. Qualified systematic review.
Gunaratne, 2015 (135)	n-3 PUFA supplementation of pregnant and/or lactating women	Little effect of maternal marine n-3 PUFA supplementation during pregnancy and/or breast feeding for the reduction of allergic disease in the children.
Hanson, 2020 (98)	Long-chain n-3 PUFA, n-6 PUFA, total PUFA	Increasing long-chain n-3 PUFA has little or no effect on cancer diagnosis, cancer death, or breast cancer diagnosis; increasing ALA has little or no effect on cancer death. Increasing total PUFA may very slightly increase cancer risk, offset by small protective effects on CVD. Qualified systematic review.
Hooper, 2020 (16)	Effects of total fat intake on body fatness in adults	Reducing total fat intake results in small reductions in body fatness. Reducing fat intake to a greater extent results in greater weight reduction. Qualified systematic review.
Hooper, 2020 (49)	SFA	Reducing SFA intake for at least 2 years causes reduction in combined cardiovascular events. Replacing the energy from SFA with PUFA or carbohydrate appears to be useful strategies, while effects of replacement with monounsaturated fat are unclear. Qualified systematic review.
Mensink, 2016 (22)	SFA	Replacing SFA by PUFA and MUFA improves blood lipoprotein profile (e.g. decreasing LDL). Lauric, myristic, and palmitic acids increase LDL whereas stearic acid is neutral (compared with carbohydrate). Qualified systematic review.
Naude, 2018 (17)	Effects of total fat intake on bodyweight in children	No high-quality evidence with which to answer this question. Small reductions in BMI, total- and LDL at some time points with lower fat intake compared to controls. Qualified systematic review.
Newberry, 2016 (127)	n-3 PUFA	n-3 PUFA supplementation increases length of gestation and birth weight but has no effects on peripartum maternal or infant health outcomes, or gestational hypertension, peripartum depression, or postnatal growth. Qualified systematic review.
Reynolds, 2022 (54)	SFA and TFA	Higher intakes of SFA and TFA were associated with increased all-cause mortality. CHD incidence reduced with a 5% energy replacement with PUFA, plant MUFA, and slowly digested carbohydrates. Qualified systematic review.
Schwab, 2014 (26)	Amount and types of fat	Replacement of SFA by PUFA or MUFA lowers LDL. Total fat intake may increase body weight. Replacement of SFA by PUFA decreases risk of CVD. Inconclusive evidence regarding total fat, as well as n-3 PUFA, and T2D. No clear evidence regarding amount or type of fat for cancer. Qualified systematic review.
Te Morenga, 2017 (55)	SFA and TFA in children and adolescents	Advice to reduce SFA intake of children results in a significant reduction in total and LDL-C as well as diastolic BP without evidence of adverse effects on growth and development. Guidelines for children/adolescents should recommend diets low in SFA. Qualified systematic review.
Wolfram, 2015 (31)	Types of fat	Reducing SFA and increasing PUFA reduce LDL and risk of CHD. TFA increases risk of dyslipoproteinemia and CHD. LCn-3 PUFA reduces TG, and potentially risk of hypertension and CHD. Qualified systematic review.

**Table 2:** Included search hits

First author, year (reference)	Exposure	Main findings/conclusions	AMSTAR2-NNR rating
***Cardiovascular disease and blood lipids***			
Bendsen, 2011 (56)	TFA	No firm conclusion can be drawn regarding risk of CHD for ruminant compared to industrial TFA	Moderate confidence
Marklund, 2019 (37)	n-6 PUFA	Circulating linoleic acid is inversely associated with CVD, cardiovascular mortality, and ischemic stroke	High confidence
Panth, 2018 (51)	Medium-chain FA	Compared to long-chain SFA, medium-chain SFA increases HDL but has no differential effect on LDL	High confidence
Verneque, 2020 (57)	Ruminant and industrial TFA	Ruminant TFA may increase LDL cholesterol more than industrial TFA. Effects are different in men and women	High confidence
Trieu, 2021 (52)	15:0, 17:0, t16:1n-7	Levels of 15:0 and 17:0, but not t16:1n-7, in plasma or adipose tissue was inversely associated with total CVD	High confidence
Gencer, 2021 (41)	n-3 PUFA	n-3 PUFA supplements increase the risk of atrial fibrillation	High confidence
Casula, 2020 (32)	n-3 PUFA	n-3 PUFA supplements reduce risk of cardiac mortality, MACE and MI. Benefits are higher in secondary prevention and with doses over 1 g/d	Low confidence
Lombardi, 2020 (36)	n-3 PUFA	n-3 PUFA supplements reduce risk of cardiovascular events. Higher doses are more effective, but also increase risk of bleeding and atrial fibrillation	Moderate confidence
Ueno, 2019 (39)	n-3 PUFA	n-3 PUFA supplements does not influence the risk of stroke	Low confidence
Sekikawa, 2019 (42)	n-3 PUFA	n-3 PUFA supplements may slow progression of atherosclerosis	High confidence
Lotfi, 2021 (43)	MUFA	No association with CVD mortality	Moderate confidence
***Hypertension and blood pressure***			
Verneque, 2020 (57)	Ruminant and industrial TFA	No differential effect of ruminant and industrial TFA on systolic or diastolic blood pressure	High confidence
Schwingshackl, 2011 (58)	MUFA	Compared to low-MUFA diets (<12E%), high-MUFA diets (>12E%) lowers both systolic and diastolic blood pressure	High confidence
***Diabetes, glucose and insulin***			
Liu, 2019 (67)	PUFA	No clear effect of n-3 or -6 supplementation on preclinical or clinical type 1 diabetes in children	Moderate confidence
Imamura, 2018 (64)	Dairy fat biomarkers	Circulating dairy fat biomarkers are associated with lower incidence of type 2 diabetes	Moderate confidence
Neuenschwander, 2020 (62)	SFA, vegetable fat, PUFA, ALA, n-3 PUFA	Amount or type of fat not associated with incidence of type 2 diabetes	High confidence
Verneque, 2020(57)	Ruminant and industrial TFA	No differential effect of ruminant and industrial TFA on glucose, insulin, and insulin sensitivity	High confidence
Aronis, 2012(65)	TFA	Higher compared to lower intake of TFA had no effect on glucose or insulin	Critically low confidence
***Cancer***			
Alexander, 2015(69)	n-3 PUFA	No association with prostate cancer, either self-reported intake or biomarkers	Critically low confidence
Anjom-Shoae, 2020(92)	TFA	No association with breast cancer	Low confidence
Aucoin, 2017(70)	n-3 PUFA	No association with prostate cancer	High confidence
Brennan, 2017(94)	Total fat, SFA	SFA, but not total fat, positively associated with breast cancer mortality	Critically low confidence
Cao, 2016(93)	Total fat, SFA, MUFA, PUFA, n-3 PUFA, n-6 PUFA	Total fat positively associated with breast cancer	Low confidence
Chen, 2015 (91)	n-3 PUFA	No association with colorectal cancer	Critically low confidence
Fu, 2015 (71)	n-3 PUFA	ALA inversely and DHA positively associated with prostate cancer. EPA not associated. DHA in blood positively associated, ALA and EPA not associated	Low confidence
Gao, 2015 (81)	n-3 PUFA	No association with liver cancer	Low confidence
Han, 2017 (80)	Total fat, animal fat, vegetable fat	Total and animal fat positively associated with non-Hodgkin lymphoma, vegetable fat not associated	Critically low confidence
Han, 2015 (82)	SFA, MUFA, PUFA, total fat	No association with gastric cancer in one cohort, total fat and SFA positively associated in case-control studies and PUFA inversely associated	Low confidence
Jiang, 2015 (85)	SFA, PUFA, total fat	Total fat inversely associated with endometrial cancer	Low confidence
Kim, 2020 (68)	n-6 PUFA	Blood levels of n-6 PUFA inversely associated with any cancer, self-reported intake not associated	Low confidence
Kim, 2018 (88)	SFA, MUFA, PUFA, total fat	No association with colorectal cancer	Critically low confidence
Kim, 2021 (40)	Total PUFA, SFA	PUFA associated with lower cancer mortality, SFA associated with higher cancer mortality	High confidence
Liu, 2021 (72)	n-3 PUFA	ALA and n-6 PUFA not associated with prostate cancer, total n-3 PUFA positively associated. Marine n-3 PUFA inversely associated with breast cancer	Critically low confidence
Liu, 2011 (89)	Total fat, animal fat, plant fat	No association with colorectal cancer	Critically low confidence
Nguyen, 2021 (87)	Total fat, SFA, MUFA, PUFA, n-3 PUFA, n-6 PUFA	No association with colorectal cancer	Critically low confidence
Nindrea, 2019 (96)	n-3/n-6 PUFA ratio	No association with breast cancer	Critically low confidence
Noel, 2014 (74)	n-3 PUFA	No association with skin cancer	Critically low confidence
Qiu, 2016 (78)	SFA, MUFA, PUFA, total fat	No association with ovarian cancer	Critically low confidence
Ruan, 2020 (75)	SFA, MUFA, PUFA, total fat	No association with skin cancer	Low confidence
Sadeghi, 2019 (79)	Total fat, TFA, SFA, MUFA	Total fat and TFA positively associated with ovarian cancer	Critically low confidence
Shen, 2015 (76)	Total fat	No association with pancreatic cancer	Critically low confidence
Shen, 2012 (90)	n-3 PUFA	No association with colorectal cancer	Critically low confidence
Wang, 2019 (83)	SFA, MUFA, PUFA, total fat	No associations with bladder cancer in cohorts, total fat positively associated in meta-analysis including case-control studies	Critically low confidence
Wu, 2015 (86)	SFA, MUFA, PUFA	SFA and MUFA, but not PUFA, inversely associated with endometrial cancer	Low confidence
Xu, 2015 (73)	SFA, MUFA, PUFA, total fat	No association with prostate cancer	Low confidence
Yao, 2015 (77)	SFA, MUFA, PUFA	No association with pancreatic cancer	Low confidence
Zhao, 2016 (84)	Total fat	No association with endometrial cancer	Low confidence
Zheng, 2013 (95)	n-3 PUFA	Inverse association with breast cancer	Low confidence
Lv, 2021 (97)	PUFA	No association with lung cancer	Low confidence
Lotfi, 2021 (43)	MUFA	No association with cancer mortality	Low confidence
***Osteoporosis and bone health***			
Mozaffari, 2018 (103)	Total fat, SFA, MUFA	SFA and animal-derived MUFA positively associated with risk of bone fracture	Critically low confidence
Shen, 2017 (100)	n-3 PUFA	n-3 PUFA may affect some markers of bone turnover	Critically low confidence
Sadeghi, 2019 (101)	n-3 PUFA	Inverse association with hip fracture	Critically low confidence
Orchard, 2012 (99)	n-3 PUFA	Evidence too limited to draw firm conclusions on potential effects on skeletal health	Critically low confidence
***Other health outcomes and risk factors***			
Huang, 2020 (123)	n-3 PUFA	There were minor benefits for muscle mass gain and timed up and go performance. n-3 PUFA may improve ‘time up and go test’ and increase muscle mass, but not handgrip strength, walking speed or one-repetition maximum leg strength	Medium confidence
Bird, 2021 (124)	n-3 PUFA	n-3 PUFA may increase lean body mass and maximal voluntary capacity, but not handgrip strength	Low confidence
Zhang, 2022 (125)	n-3 PUFA, n-6 PUFA	Dietary n-3 PUFA, but not n-6 PUFA, inversely associated with sarcopenia	Low confidence
Fonseca, 2014 (126)	Types of fat	No conclusion can be drawn for COPD	Critically low Confidence
Falsig, 2019 (114)	n-3 PUFA	n-3 PUFA may improve semen quality parameters	Moderate confidence
Kdekian, 2020 (121)	Higher versus lower fat	Exchange of carbohydrates for fats reduces postprandial glucose and PPI and increases postprandial TG.	Critically low confidence
Pase, 2011(115)	n-3 PUFA	n-3 PUFA supplementation reduce arterial stiffness	Low confidence
Wang, 2012 (116)	n-3 PUFA	n-3 PUFA supplementation improves endothelial function	Critically low confidence
Li, 2014 (117)	n-3 PUFA	n-3 PUFA supplementation lowers CRP, IL-6 and TNF-α	High confidence
Winters-van Eekelen, 2021 (108)	Total fat, SFA, unsaturated fat	Unsaturated fat decrease liver fat content compared to SFA. Replacing fat with carbohydrate was not effective	Low confidence
Candido, 2020 (122)	SFA, PUFA	May affect endotoxemia in acute, but not longitudinal, settings	High confidence
Heshmati, 2019 (118)	n-3 PUFA	Improved some markers of oxidative stress	Low confidence
***Total mortality***			
Kim, 2021 (40)	SFA, MUFA, PUFA, total fat	A 5% increase in energy from PUFA was associated with 5% and 4% lower mortality from CVD and cancer, respectively. A 1% energy increment in TFA was associated with 6% higher risk of mortality from all-causes and CVD. There was a non-linear association between dietary SFA and all-cause mortality showing a significant increased risk up to 11% of energy from SFA. The risk of cancer mortality increased by 4% for every 5% increase in energy from SFA	High confidence
Lotfi, 2021 (43)	MUFA	No significant association between MUFA intake and risk of CVD mortality or cancer mortality. An additional 5% of energy from MUFA was associated with a 3% reduced risk of all-cause mortality, but not with CVD and cancer mortality	High confidence
Trieu, 2021 (52)	15:0, 17:0, t16:1n-7	No association with all-cause mortality. Higher levels of 15:0 and 17:0 were associated with lower CVD risk	High confidence
***Mother and child health***			
Ambrozej, 2021 (140)	MFGM	MFGM-supplemented formulas may improve some aspects of psychomotor development compared to standard formula	Critically low confidence
Kar, 2016 (131)	n-3 PUFA	Maternal n-3 PUFA supplementation reduces the risk of preterm delivery	Critically low confidence
Li, 2018 (132)	n-3 PUFA	Maternal n-3 PUFA supplementation has no or little effect on offspring body composition	Critically low confidence
Ren, 2021 (133)	PUFA and TFA	Maternal n-3 PUFA supplementation has no or little effect on offspring body weight or BMI at ages 0–4 years and 5–10 years. Blood/tissue levels of TFA are associated with lower birth weight in observational studies	High confidence
Wu, 2019 (139)	PUFA, ruminant TFA	Exposure to n-3 PUFA in early life not associated with development of allergic disease (eczema, asthma, wheeze, allergic rhinitis, sensitization). Exposure to vaccenic acid inversely associated with development of eczema whereas exposure to linoleic acid was positively associated.	Low confidence
Amirani, 2020 (134)	n-3 PUFA	n-3 PUFA supplement during pregnancy increase HDL cholesterol and decrease CRP, but has no effect on other lipid or inflammation markers or glycemia	Critically low confidence
Muley, 2015 (136)	n-3 PUFA	n-3 PUFA supplements to children had no effect on primary prevention of asthma	Critically low confidence
Waidyatillake, 2018 (138)	n-3 PUFA, n-6 PUFA	Insufficient evidence to determine if PUFA in breast milk is associated with development of allergic disease in children	Low confidence
Verfuerden, 2020 (130)	Long-chain PUFA	Infant formula supplemented with long-chain PUFA has no effect on long-term cognitive function in childhood	High confidence

**Table 3:** Excluded search hits

Title	First author	Year	PMID	Reason for excluding
A Meta-Analysis of Randomized Controlled Trials and Prospective Cohort Studies of Eicosapentaenoic and Docosahexaenoic Long-Chain Omega-3 Fatty Acids and Coronary Heart Disease Risk	Alexander	2017	28062061	Covered by qSR (Abdelhamid 2020)
Very-low-carbohydrate ketogenic diet v. low-fat diet for long-term weight loss: a meta-analysis of randomised controlled trials	Bueno	2013	23651522	Covered by qSR (Schwab 2014)
N-3 long-chain polyunsaturated fatty acids and risk of all-cause mortality among general populations: a meta-analysis	Chen	2016	27306836	Covered by qSR (Abdelhamid 2020)
Can dietary saturated fat be beneficial in prevention of stroke risk? A meta-analysis	Cheng	2016	26979840	Covered by qSR (Hooper 2020, DGAC 2020, Mensink 2016)
Association between fish consumption, long-chain omega 3 fatty acids, and risk of cerebrovascular disease: systematic review and meta-analysis	Chowdhury	2012	23112118	Covered by qSR (Abdelhamid 2020, Balk 2016)
Association of dietary, circulating, and supplement fatty acids with coronary risk: a systematic review and meta-analysis	Chowdhury	2014	24723079	Covered by qSR (Abdelhamid 2020, DGAC 2020, Balk 2016)
A systematic review of the effect of dietary saturated and polyunsaturated fat on heart disease	Clifton	2017	29174025	Covered by qSR (Abdelhamid 2020, DGAC 2020, Balk 2016)
Circulating fatty acids and prostate cancer risk: individual participant meta-analysis of prospective studies	Crowe	2014	25210201	No SR
Fish consumption, omega-3 fatty acids and risk of heart failure: a meta-analysis	Djoussé	2012	22682084	Covered by qSR (Abdelhamid 2020)
Review of the evidence for the potential impact and feasibility of substituting saturated fat in the New Zealand diet	Foster	2013	23895475	Covered by qSR (Abdelhamid 2020, DGAC 2020, Balk 2016)
Is there a linear relationship between the dose of ruminant trans-fatty acids and cardiovascular risk markers in healthy subjects: results from a systematic review and meta-regression of randomised clinical trials	Gayet-Boyer	2014	25345440	Covered by qSR (Brouwer 2016)
Clinical Outcomes of Dietary Replacement of Saturated Fatty Acids with Unsaturated Fat Sources in Adults with Overweight and Obesity: A Systematic Review and Meta-Analysis of Randomized Control Trials	Hannon	2017	28768248	Covered by qSR (Abdelhamid 2020)
Evidence from prospective cohort studies does not support current dietary fat guidelines: a systematic review and meta-analysis	Harcombe	2017	27697938	Covered by qSR (Abdelhamid 2020, DGAC 2020, Balk 2016)
Effect of reducing total fat intake on body weight: systematic review and meta-analysis of randomised controlled trials and cohort studies	Hooper	2012	23220130	Covered by qSR (Schwab 2014)
Dietary fat and fatty acid intake and epithelial ovarian cancer risk: evidence from epidemiological studies	Hou	2015	26515595	Oldest, least number of cohorts (all but 1 covered in the others)
Effects of low-carbohydrate diets versus low-fat diets on metabolic risk factors: a meta-analysis of randomized controlled clinical trials	Hu	2012	23035144	Covered by Schwingshackl 2013
Omega-3 and omega-6 polyunsaturated fatty acids and metabolic syndrome: A systematic review and meta-analysis	Jang	2020	31010701	MetS is a composite with different definitions, components covered by other SR
Effect of omega-3 fatty acids supplementation on anthropometric indices in children and adolescents: A systematic review and meta-analysis of randomized controlled trials	Jazayeri	2020	33066870	Issue Covered by qSR (Naude 2018)
Dietary saturated fat intake and risk of stroke: Systematic review and dose-response meta-analysis of prospective cohort studies	Kang	2020	31791641	Covered by qSR (Hooper 2020, DGAC 2020)
Do omega-3 polyunsaturated fatty acids reduce risk of sudden cardiac death and ventricular arrhythmias? A meta-analysis of randomized trials	Khoueiry	2013	23714269	Covered by qSR (Abdelhamid 2020, Balk 2016)
Efficacy of Dietary Supplements to Reduce Liver Fat	Kilchoer	2020	32751906	Covered by Winters-van Eekelen E. 2021
Current information and Asian perspectives on long-chain polyunsaturated fatty acids in pregnancy, lactation, and infancy: systematic review and practice recommendations from an early nutrition academy workshop	Koletzko	2014	25227906	Asian perspective
Omega 3 Fatty acids and cardiovascular outcomes: systematic review and meta-analysis	Kotwal	2012	23110790	Covered by qSR (Abdelhamid 2020)
Atopy risk in infants and children in relation to early exposure to fish, oily fish, or long-chain omega-3 fatty acids: a systematic review	Kremmyda	2011	19997989	Covered by SR (Gunaratne 2015)
Long-chain omega-3 polyunsaturated fatty acids and risk of stroke: a meta-analysis	Larsson	2012	23179632	Covered by qSR (Abdelhamid 2020)
Consumption of Fish and ω-3 Fatty Acids and Cancer Risk: An Umbrella Review of Meta-Analyses of Observational Studies	Lee	2020	32488249	Umbrella review
The cardiovascular benefits of indiscriminate supplementation of omega-3 fatty acids; meta-analysis and decision-making approach	Leshno	2018	29171335	Covered by qSR (Abdelhamid 2020)
A review of the effect of omega-3 polyunsaturated fatty acids on blood triacylglycerol levels in normolipidemic and borderline hyperlipidemic individuals	Leslie	2015	26048287	Covered by qSR (Abdelhamid 2020)
DietaryFish and Long-Chain n-3 Polyunsaturated Fatty Acids Intake and Risk of Atrial Fibrillation: A Meta-Analysis	Li	2017	28850090	Covered by qSR (Abdelhamid 2020)
Effects of low-fat compared with high-fat diet on cardiometabolic indicators in people with overweight and obesity without overt metabolic disturbance: a systematic review and meta-analysis of randomised controlled trials	Lu	2018	29212558	Covered by qSR (Hooper 2020)
Use of supplemental long-chain omega-3 fatty acids and risk for cardiac death: An updated meta-analysis and review of research gaps	Maki	2017	28818347	Covered by qSR (Abdelhamid 2020)
Effects of low-carbohydrate diets v. low-fat diets on body weight and cardiovascular risk factors: a meta-analysis of randomised controlled trials	Mansoor	2016	26768850	Covered by qSR (Hooper 2020)
N-3 polyunsaturated fatty acids to prevent atrial fibrillation: updated systematic review and meta-analysis of randomized controlled trials	Mariani	2013	23525440	Covered by qSR (Abdelhamid 2020)
Long-chain omega-3 fatty acids eicosapentaenoic acid and docosahexaenoic acid and blood pressure: a meta-analysis of randomized controlled trials	Miller	2014	24610882	Covered by qSR (Abdelhamid 2020)
Dietary intake of fish, n-3 polyunsaturated fatty acids, and risk of inflammatory bowel disease: a systematic review and meta-analysis of observational studies	Mozaffari	2020	30680455	Covered by qSR (Ajabnoor 2021)
ALA, fatty fish or marine n-3 fatty acids for preventing DM?: a systematic review and meta-analysis	Muley	2014	24828061	Newer data to be used
High-Fat Ketogenic Diets and Physical Performance: A Systematic Review	Murphy	2021	32865567	Not relevant
High Dietary Saturated Fat is Associated with a Low Risk of Intracerebral Hemorrhage and Ischemic Stroke in Japanese but not in Non-Japanese: A Review and Meta-Analysis of Prospective Cohort Studies	Muto	2018	29269706	Covered by qSR (Abdelhamid 2020, DGAC 2020, Balk 2016)
Protective Effect of Omega-3 Fatty Acids in Fish Consumption Against Breast Cancer in Asian Patients: A Meta-Analysis	Nindrea	2019	30803190	Food level; only Asian population
Whole-Fat or Reduced-Fat Dairy Product Intake, Adiposity, and Cardiometabolic Health in Children: A Systematic Review	O’Sullivan	2020	32119732	Issue Covered by qSR (Naude 2018); food level
Nutritional interventions or exposures in infants and children aged up to 3 years and their effects on subsequent risk of overweight, obesity and body fat: a systematic review of systematic reviews	Patro-Gołąb	2016	27749991	Umbrella review
Omega 3 fatty acids in cardiovascular disease risk factors: An updated systematic review of randomised clinical trials	Rangel-Huerta	2018	28601400	Covered by qSR (Hooper 2020, DGAC 2020, Mensink 2016)
Omega-3 long-chain polyunsaturated fatty acids supplementation on inflammatory biomakers: a systematic review of randomised clinical trials	Rangel-Huerta	2012	22591890	Covered by qSR (Abdelhamid 2020)
Effect of n-3 long-chain polyunsaturated fatty acids during the perinatal period on later body composition	Rodríguez	2012	22591886	Covered by Li 2018
Role of fatty acids and micronutrients in healthy ageing: a systematic review of randomised controlled trials set in the context of European dietary surveys of older adults	Ruxton	2016	26286890	Diverse outcomes, covered by other SRs
Omega-3 long-chain polyunsaturated fatty acids and fish oil supplementation during pregnancy: which evidence?	Saccone	2016	26382010	Covered by qSR (Newberry and Delgado-Noguera)
Omega-3 long-chain polyunsaturated fatty acids to prevent preterm birth: a systematic review and meta-analysis	Saccone	2015	25730231	Covered by Kar 2016
Dietary Intervention for Overweight and Obese Adults: Comparison of Low-Carbohydrate and Low-Fat Diets. A Meta-Analysis	Sackner-Bernstein	2015	26485706	Covered by qSR (Hooper 2020)
Diet and body fat in adolescence and early adulthood: a systematic review of longitudinal studies	Schneider	2017	28538925	Covered by qSR (Hooper 2020)
Comparison of effects of long-term low-fat versus high-fat diets on blood lipid levels in overweight or obese patients: a systematic review and meta-analysis	Schwingshackl	2013	24139973	Covered by qSR (Hooper 2020)
Monounsaturated fatty acids, olive oil and health status: a systematic review and meta-analysis of cohort studies	Schwingshackl	2014	25274026	Covered by qSR (Hooper 2020)
Statin Use Mitigate the Benefit of Omega-3 Fatty Acids Supplementation-A Meta-Regression of Randomized Trials	Sethi	2016	25036814	Covered by qSR (Abdelhamid 2020)
A Systematic Review and Meta-Analysis Comparing Heterogeneity in Body Mass Responses Between Low-Carbohydrate and Low-Fat Diets	Smith	2020	32959516	Issue Covered by qSR (Naude 2018)
Palm Oil Consumption Increases LDL Cholesterol Compared with Vegetable Oils Low in Saturated Fat in a Meta-Analysis of Clinical Trials	Sun	2015	25995283	Food level data
Effect of low-fat diet interventions versus other diet interventions on long-term weight change in adults: a systematic review and meta-analysis	Tobias	2015	26527511	Covered by qSR (Hooper 2020)
Fish consumption, dietary long-chain n-3 fatty acids, and risk of type 2 diabetes: systematic review and meta-analysis of prospective studies	Wallin	2012	22442397	Newer data to be used
Fish, long-chain omega-3 polyunsaturated fatty acids consumption, and risk of all-cause mortality: a systematic review and dose-response meta-analysis from 23 independent prospective cohort studies	Wan	2017	28802305	Covered by qSR (Abdelhamid 2020)
Breast milk n-3 long-chain polyunsaturated fatty acids and blood pressure: an individual participant meta-analysis	van Rossem	2021	32564149	Not an SR
Whole milk compared with reduced-fat milk and childhood overweight: a systematic review and meta-analysis	Vanderhout	2020	31851302	Food level
Plant-derived polyunsaturated fatty acids and markers of glucose metabolism and insulin resistance: a meta-analysis of randomized controlled feeding trials	Wanders	2019	30899527	Covered by qSR (Ajabnoor 2021)
The Effect of Supplementation of Long-Chain Polyunsaturated Fatty Acids During Lactation on Neurodevelopmental Outcomes of Preterm Infant From Infancy to School Age: A Systematic Review and Meta-analysis	Wang	2016	27318249	Covered by qSR (Newberry and Delgado-Noguera)
Effects of macronutrient intake in obesity: a meta-analysis of low-carbohydrate and low-fat diets on markers of the metabolic syndrome	Willems	2021	32885229	MetS is a composite with different definitions, components covered by other SR
Effects of low-fat diet on serum lipids in premenopausal and postmenopausal women: a meta-analysis of randomized controlled trials	Wu	2014	23736858	Covered by Schwingshackl 2013, only women
Omega-3 fatty acids and incident type 2 diabetes: a systematic review and meta-analysis	Wu	2012	22591895	Newer data to be used
The effects of low-fat, high-carbohydrate diets versus low-carbohydrate, high-fat diets on weight, blood pressure, serum liquids and blood glucose: a systematic review and meta-analysis	Yang	2022	34168293	Weight loss diets
Efficacy of Omega-3 Polyunsaturated Fatty Acids Supplementation in Managing Overweight and Obesity: A Meta-Analysis of Randomized Clinical Trials	Zhang	2017	28112774	Covered by qSR (Hooper 2020)
The limited effect of omega-3 polyunsaturated fatty acids on cardiovascular risk in patients with impaired glucose metabolism: a meta-analysis	Zheng	2014	24342751	Covered by qSR (Abdelhamid 2020, Balk 2016)
Marine N-3 polyunsaturated fatty acids are inversely associated with risk of type 2 diabetes in Asians: a systematic review and meta-analysis	Zheng	2012	22984522	Newer data to be used
Dietary total fat, fatty acids intake, and risk of cardiovascular disease: a dose-response meta-analysis of cohort studies	Zhu	2019	30954077	Covered by qSR (Abdelhamid 2020, DGAC 2020, Balk 2016)

## Physiology

Triglycerides are hydrolyzed by lipases in the gut to monoglycerides and fatty acids, which together with bile salts, lysophospholipids, and unesterified cholesterol form mixed micelles from which the digested lipids are absorbed in the small intestine. Fats are not soluble in water and are transported in the blood as lipoprotein particles. The core of the lipoprotein particles is formed by triglycerides and esterified cholesterol, whereas the surface is composed of free cholesterol, phospholipids, and proteins.

Cholesterol, in addition to being consumed through the diet (meat, eggs, and dairy being the primary sources), is synthesized in the body and is used for the production of bile acids, steroid hormones, vitamin D, and for cell membrane structures. Importantly, the synthesis of cholesterol and its metabolites occur intracellularly and are highly regulated. About 1 g of cholesterol is synthesized in human adults every day, and this is 3–4 times the amount absorbed from the average adult Nordic diet. The fractional absorption of cholesterol is reduced when the dietary intake increases. On average, 40–50% of dietary cholesterol is absorbed, but absorption varies between individuals and can range from 20 to 80%.

The human body is capable of synthesizing SFAs and MUFAs – including n-7 and n-9 series MUFAs – from acetate, but n-3 and n-6 series PUFAs are essential and required from the diet. Linoleic acid (n-6, LA) and α-linolenic acid (n-3, ALA) are the two essential fatty acids that must be provided in the diet because the human body lacks the enzymes ∆12- and ∆15-desaturase that are capable of introducing double bonds at the n-6 and n-3 positions. Linoleic acid and ALA are metabolized (desaturated and elongated) further in the body by the same enzymes; however, the n-3 series fatty acids have a higher affinity for the enzymes. Theoretically, an imbalance between dietary intakes of LA and ALA might thus influence the further metabolism to more long-chain and unsaturated n-6 and n-3 fatty acids; however, the total intake of each of the n-6 and n-3 fatty acids appears more important than the ratio, as long as basic dietary requirements are covered. Linoleic acid may be metabolized to arachidonic acid (AA, 20:4n-6) whereas ALA may be metabolized to EPA (20:5n-3) and DHA (22:6n-3). However, these conversions are regulated and there are considerable interindividual variations (4–7) with unknown clinical relevance. The n-6 and n-3 PUFAs, particularly the long-chain molecules, are important structural components of cell membranes, for fluidity, permeability, activity of membrane-bound enzymes and receptors, and signal transduction.

## Assessment of nutrient status

The assessment of dietary intake of total fat and fatty acids mainly relies on self-reported data (e.g. food-frequency questionnaires, dietary records and recalls). There is no validated objective biomarker for total fat intake. However, the relative proportion of some individual fatty acids (primarily PUFAs) in blood/tissues is generally accepted as valid and objective biomarkers of intake. Examples include LA and DHA, but the odd-chain fatty acids 15:0 and 17:0 are also commonly used to reflect intake of fat from ruminants (primarily dairy fat). However, there is an ongoing discussion of the utility of odd-chain fatty acids to reflect dietary intake of dairy fat as there are other potential contributors, for example, fish intake and endogenous synthesis from dietary fiber, and intervention studies do not always show altered levels in relation to altered intakes (8–10). Fatty acid biomarkers obtained from blood/tissues are not quantitative but may be used to rank individuals from higher to lower dietary intake and are a useful and valuable complement when assessing diet-health relationships.

## Diet intake in Nordic and Baltic countries

In the Nordic countries, the intake of total fat (as a percentage of total energy) is highest in Finland (men: 38.7%, women: 37.7%) followed by Iceland (men: 36.7%, women: 35.9%) and Denmark (men: 36%, women: 36%). Total fat intake is slightly lower in Sweden (men: 34.0%, women: 34.4%) and Norway (men: 34%, women: 34%). In the Baltic countries, total fat intake is higher in Lithuania (men: 43.7%, women: 42.1%) and Latvia (men: 40.6%, women: 40.8%) than in Estonia (men: 34.9%, women: 34.7%). In Iceland, intakes of total fat, SFAs, and MUFAs increase with age; however, these gradients are not observed in the other countries. Intake of SFAs is above the recommendation of 10% of energy in all countries, ranging from 13% in Norway and Sweden to ~15% in Denmark and Finland, with intakes slightly higher in men than women (11). Intake of PUFA is highest in Finland (men: 6.8%, women: 6.9%), followed by Norway (men: 6.3%, women: 6.2%), Iceland (men: 5.9%, women: 6.0%), and Estonia (men: 6.0%, women: 5.9%), with slightly lower intakes in Sweden (men: 5.5%, women: 5.7%) and Denmark (men: 5.5%, women: 5.6%). Intakes of MUFA range from ~11.5% in Iceland to ~14.5% in Finland. Intake of cholesterol is lowest in Finland (men: 299 mg, women: 231 mg) and highest in Lithuania (men: 415 mg, women: 307 mg).

## Health outcomes relevant for Nordic and Baltic countries

### Deficiencies

Deficiency of the essential fatty acids, LA and ALA, in adults is very rare (12). Reported cases have been associated with chronic diseases or prolonged parenteral or enteral nutrition either without fat or very low in fat. The minimum requirements of LA and ALA remain unknown, but diets containing 1 E% LA and 0.2–0.5 E% ALA appear to be sufficient to prevent the development of essential fatty acid deficiency (12). Combined deficiency of LA and ALA leads to increased formation of the PUFA C20:3 n-9 and an increased C20:3 n-9/C20:4 n-6 ratio. It has not been confirmed, however, that this ratio is a useful indicator of deficiency in humans although it is used as such (12). Clinical symptoms of deficiency (skin changes and growth retardation) have been found in healthy, new-born babies fed for 2–3 months with a diet low (<1 E%) in LA.

Diets low in total fat may compromise the intake and absorption of fat-soluble vitamins.

### Toxicities

In humans, high intakes of PUFA can potentially result in adverse effects including increased lipid peroxidation, impaired immune function, and increased bleeding tendency (13). Intakes of n-6 fatty acids (LA) up to around 10 E% are considered safe (13, 14). The EFSA concluded that combined long-term supplemental intakes of EPA and DHA up to about 5 g per day did not appear to increase the risk of spontaneous bleeding episodes or bleeding complications or to affect glucose homeostasis, immune function, or lipid peroxidation provided that the oxidative stability of the n-3 long-chain PUFA was guaranteed (15).

### Nutrient-related chronic diseases in Nordic and Baltic countries

#### Obesity and body weight

Here, we present findings from two qSRs commissioned by the WHO, one in adults (16) and one in children (17).

To understand how dietary fat relates to body weight and body fatness in the general population, a qSR on randomized controlled trials (RCTs) in adults was performed until October 2019 (16). The 37 studies included 57,079 participants who were randomized to a lower fat (≤30 E%) versus higher fat (>30 E%) diet, without the intention to reduce weight in any participants for at least 6 months. They found that the effect of eating less fat (compared with higher fat intake) was a mean body weight reduction of 1.4 kg (95% confidence interval [CI]: −1.7 to −1.1 kg). The data suggested that there was a greater weight loss in people with higher body mass index (BMI) at baseline. The reduction in body weight was reflected in small reductions in low-density lipoprotein cholesterol (LDL-C) (−0.13 mmol/L [−0.21 to −0.05]) and total cholesterol (−0.23 mmol/L [−0.32 to −0.14]), with little or no effect on high-density lipoprotein cholesterol (HDL-C) (−0.02 mmol/L [−0.03 to 0.00]), triglycerides (0.01 mmol/L [−0.05 to 0.07]), systolic (−0.75 mm Hg [−1.42 to −0.07]) or diastolic blood pressure (−0.52 mm Hg [−0.95 to −0.09]), all GRADE high-quality evidence, or quality of life (0.04 [0.01–0.07]), on a scale of 0–10, GRADE low-quality evidence. The authors concluded that there was a consistent, stable but small effect of low fat intake on body fatness in adults: slightly lower weight, BMI, waist circumference, and percentage body fat compared with higher fat arms. Greater fat reduction, lower baseline fat intake, and higher baseline BMI were all associated with greater reductions in weight. There was no evidence of adverse effects related to serum lipids, blood pressure, or quality of life, but rather of small benefits or no effect.

The WHO also commissioned an SR and meta-analysis to study the population impact of total fat intake in the development of obesity in children (17). The objective was to assess the effects and associations of total fat intake on measures of weight and body fatness in children and young people not aiming to lose weight. The study included RCTs in children aged 2–18 years randomized to a lower fat (30 E%) versus usual or moderate-fat diet (greater than 30 E%). Prospective cohort studies were also included if they related baseline total fat intake to weight or body fatness at least 12 months later. A total of 24 RCTs (*n* = 1,054 randomized) were included in addition to 21 prospective cohort studies (about 25,059 children completed). Meta-analyses were impossible; only one RCT reported the same outcome at each time point range for all outcomes; and the cohort studies were too heterogeneous to combine. The authors reported imprecision and poor reporting in the studies which limited their confidence of the findings and reduced the applicability of the data. Data from RCTs showed that in one RCT, dietary counseling to lower total fat intake found that the intervention may make little or no difference to weight compared with usual diet at 12 months (mean difference [MD]: −0.50 kg [−1.78 to 0.78]; *n* = 620; low-quality evidence) and at 3 years (MD: −0.60 kg [−2.39 to 1.19]; *n* = 612; low-quality evidence). Education delivered as a classroom curriculum probably decreased BMI in children at 17 months (MD: −1.5 kg/m^2^ [−2.45 to −0.55]; one RCT; *n* = 191; moderate-quality evidence). The effects were smaller at longer-term follow-up (5 years: MD: 0 kg/m^2^ [−0.63 to 0.63]; *n* = 541; 7 years; MD: −0.10 kg/m^2^ [−0.75 to 0.55]; *n* = 576; low-quality evidence). Dietary counseling slightly decreased LDL-C at 12 months (MD: −0.12 mmol/L [−0.20 to −0.04]; one RCT; *n* = 618, moderate-quality evidence) and at 5 years (MD: −0.09 [−0.17 to −0.01]; one RCT; *n* = 623; moderate-quality evidence), compared to controls. Of the 21 cohort studies, over half suggested an association between total fat intake increases and body fatness. The heterogeneous methods and reporting across cohort studies and the low quality made it difficult to conclude on any true relationships.

The authors conclude that there is no high-quality evidence to answer the question: ‘What is the relationship between the amount of fat a child eats and their weight and body fat?’ The authors reported limited evidence from three RCTs suggesting that dietary counseling to lower total fat intake showed small reductions in BMI compared to controls.

### Concluding remarks

Overall, consistent data show that a high content of fat in the diet (>30 E%) gives a slightly higher incidence of overweight in adults in the general population. It is, however, important to emphasize that the average findings do not show the significant individual differences or differences in food culture between countries. It is also important to emphasize that the studies discussed here have not been performed to study active weight reduction of overweight individuals but were done without the intention to reduce weight. In the case of children, it was not possible to establish any conclusion based on direct empirical evidence. However, we found that the data suggest that dietary fats have some of the same effects in children as in adults.

#### Cardiovascular diseases and blood lipids

The intake of SFA and the ratio of unsaturated to SFA are considered to be important for the serum levels of LDL-C, which is causal for the process of atherosclerosis (18, 19). In westernized societies, atherosclerosis is the underlying cause of about 50% of all deaths globally (20). In the 2019 ESC/EAS Guidelines to reduce cardiovascular risk, SFAs are the dietary factor with the greatest impact on LDL-C serum levels: 0.02–0.04 mmol/L increase in LDL-C for every additional 1 E% coming from SFA (21, 22). Dietary TFA has a similar elevating effect on LDL-C to that of SFAs; however, TFA decreases HDL-C, in contrast to SFA (23). The dietary content of TFA has, however, decreased in all Nordic countries since the 1990s and is currently below 1 E% and therefore plays a minor role at the population level. Unsaturated fat-rich vegetable oils reduce LDL-C levels (−0.42 to −0.20 mmol/L) when used in substitution of SFA-rich foods like butter (24).

#### Polyunsaturated and monounsaturated fatty acids

*Systematic review on n-6 PUFAs*. We identified one SR with main focus on the effect of n-6 PUFA on CVD risk factors (25). This study found that increasing or decreasing n-6 PUFA had no direct effects on serum lipoprotein level or blood pressure and the study did not report on CVD events. However, partial replacement of SFA with n-6 PUFA decreases the risk of CVD; this was also reported by Schwab (26) and in the more recent 2020 Dietary Guidelines Advisory Committee from the United States Department of Agriculture (27) (see Section 2.3). Furthermore, DGAC (27) reported that limited evidence suggests that intake of LA, but not AA, during adulthood may be associated with lower risk of CVD, including CVD mortality.

*Qualified systematic reviews on 18–22 carbon atom n-3 PUFA*. We identified one SR and three qSRs on the effect of n-3 PUFA on CVD (28–31). None of the studies reported any definite effect of CVD events or cardiovascular risk factors. A reduction in triglyceride levels in the blood of about 15% was reported, but no effect on LDL-C, HDL-C, total cholesterol, or blood pressure. The DGAC (27) reported that total intake of n-3 PUFAs, particularly EPA and DHA from food sources, is associated with lower risk of CVD in adults (moderate evidence). Regarding types of n-3 PUFA, they report that when 20- and 22-carbon atom fatty acids (EPA, DHA, and sometimes docosapentaenoic acid (DPA), primarily from marine sources, were assessed separately from the 18-carbon atom PUFA ALA, more consistent associations with lower risk of CVD were observed.

Wolfram (31) report that there is probable evidence that an increased intake of 20- and 22-carbon atoms n-3 fatty acids lowers risk of coronary heart disease (CHD), based on prospective cohort studies. Furthermore, there is convincing evidence that it reduces the plasma triglyceride concentration.

*Results from non-qualified SRs, biomarker studies, and RCTs.* An SR from 2020 (32), based on 16 RCTs, found that supplementation of 20- and/or 22-carbon atom n-3 PUFA was associated with a significant risk reduction of cardiac mortality (odds ratio [OR]: 0.91 [0.85–0.98]), major adverse cardiovascular event (MACE; OR: 0.90 [0.82–0.99]), and myocardial infarction (OR: 0.83 [0.71–0.98]). Such studies are designed to conclude on causality, rather than associations. They use capsules with n-3 PUFA or placebo oil; because the effect of fish food cannot be controlled with placebo, obviously a placebo fish cannot be made. EPA administered alone in high dose seemed to be greater in terms of risk reduction of MACE (−25%) or myocardial infarction (−30%) (33) than the combined EPA + DHA supplementation (34, 35).

In a 2020 SR (36) performing a pairwise and network meta-analysis on 14 studies, the authors applied a cutoff value of <1 g per day for low-dose omega-3 and >1 g per day for high dose. High dose was associated with a lower risk of cardiac death (incidence rate ratio (IRR): 0.79 [0.65–0.96] vs. control). Risk of myocardial infarction was 0.71 (0.62–0.82) in high dose versus control and 0.79 (0.67–0.92) in high dose versus low dose.

In individual-level analyses in a global consortium of 30 prospective observational studies from 13 countries (37), circulating and tissue levels of LA and AA were studied. Arachidonic acid levels were not associated with higher risk of cardiovascular outcomes. Higher levels of LA were significantly associated with lower risks of total CVD, cardiovascular mortality, and ischemic stroke, with hazard ratios (HR) per interquintile range of 0.93 (0.88–0.99), 0.78 (0.70–0.85), and 0.88 (0.79–0.98), respectively, and non-significantly with lower CHD risk (0.94 [0.88–1.00]).

An individual-level pooled analysis from 2021 studied circulating levels of n-3 PUFA and risk of CVD mortality (38). Among 15 prospective cohorts with a median of 16 years of follow-up, higher compared to lower levels of EPA (HR: 0.85 [0.77–0.94]), DPA (HR: 0.87 [0.78–0.98]), and DHA (HR: 0.79 [0.72–0.88]) were associated with lower risk for CVD mortality whereas ALA was not (HR: 0.98 [0.89–1.08]).

In a 2019 SR of epidemiological studies and large-scale clinical trials on the association of dietary intake of n-3 PUFAs with stroke, the authors conclude that the effectiveness of n-3 PUFAs for secondary prevention of stroke is yet to be elucidated (39).

An SR and meta-analysis from 2021 (40) of 19 prospective cohort studies including 1,013,273 participants and 195,515 deaths found that a 5% increase in energy from PUFA was associated with 5% (relative risk (RR): 0.95 [0.91–0.98]) lower mortality from CVD.

An SR and meta-analysis from 2021 (41) of RCTs investigated the effect of marine n-3 PUFA supplementation on atrial fibrillation (AF). Seven RCTs were included (a total of 81,210 participants) with an average follow-up of 4.9 years. Use of n-3 PUFA supplements was associated with increased risk of AF (HR: 1.25 [1.07–1.46]) and the risk was greater in studies using a higher dose (HR: 1.49 [1.04–2.15] for trials testing >1 g per day and HR: 1.12 [1.03–1.22] for trials testing 1 g or less per day).

An SR and meta-analysis from 2019 (42) investigated the effects on atherosclerosis in RCTs using high-dose marine n-3 PUFA supplements. Six RCTs were included (a total of 693 participants), which were performed in patients with CHD, type 2 diabetes (T2D), and nonalcoholic fatty liver disease; however, only one was placebo-controlled. Follow-up duration ranged from 6 to 30 months and atherosclerosis was assessed in the coronary or carotid artery using various measures like carotid intima media thickness (cIMT) by ultrasound, percent change in noncalcified plaque volume by coronary computed tomography angiography (CCTA), change in normalized total atheroma volume by intravascular ultrasound (IVUS), and change in fibrous cap thickness optical coherence tomography (OCT). Overall, n-3 PUFA supplements slowed down the progression of atherosclerosis (standardized mean difference [SMD]: −1.97 [−3.01 to −0.94], *I*^2^ = 97%).

In an SR and meta-analysis from 2021 of prospective cohort studies (43), intake of MUFA (highest vs. lowest) was not associated with fatal CVD (RR: 0.95 [0.89–1.01], *I*^2^ = 37%, *n* = 17 effect sizes from 11 studies including a total of 55,437 deaths). Similarly, dose-response analyses indicated no association for each 5% additional energy intake from MUFA and CVD mortality (RR: 0.98 [0.95–1.01]).

Since 2019, some of the largest placebo-controlled double-blind studies have been published on n-3 PUFA.

In the largest RCT on n-3 PUFA ever, the VITAL trial (Vitamin D and Omega-3 Trial), 25,871 participants underwent randomization to marine n-3 PUFA at a dose of 1 g per day or placebo (44). During a median follow-up of 5.3 years, major cardiovascular events occurred in 386 participants in the n-3 PUFA group and in 419 in the placebo group (HR: 0.92; 95% CI: 0.80–1.06; *P* = 0.24), showing no difference in primary endpoints between the two groups.

In another recent RCT, the ASCEND trial (A Study of Cardiovascular Events in Diabetes), a total of 15,480 patients over the age of 40 years with diabetes and no prior CVD history at baseline were randomized to 1 g n-3 PUFA versus matching placebo (olive oil) and followed up for a mean (standard deviation [SD]) of 7.4 (1.8) years (45). The primary composite outcome did not achieve significance; 3.2% of patients in the n-3 PUFA arm developed the primary composite outcome, compared to 3.6% in the placebo group (*P* = 0.15).

One RCT has shown particularly strong effects of n-3 PUFA, the REDUCE-IT trial (Reduction of Cardiovascular Events with Icosapent Ethyl Intervention Trial) (33). In this study pure EPA (icosapent ethyl), without DHA, was used in the high dose of 4 g per day given to statin-treated patients with established CVD (or at high risk) with slightly increased triglycerides level >1.5 mmol/L and < 5.6 mmol/L. A total of 8,179 patients were randomized and followed for a median of 4.9 years. The primary endpoint (composite CVD event) occurred in 17.2% of the patients in the icosapent ethyl group, as compared with 22.0% of the patients in the placebo group (HR: 0.75; 95% CI: 0.68–0.83; *P* < 0.001). There were, however, higher rates of AF and peripheral edema among those using EPA compared to placebo (pharmaceutical-grade mineral oil). Multiple analyses and substudies have been performed showing various benefits of icosapent ethyl in separate patient groups, including reduced plaque burden (46, 47).

In another recent large RCT, the STRENGTH trial (Long-Term Outcomes Study to Assess Statin Residual Risk with Epanova in High Cardiovascular Risk Patients with Hypertriglyceridemia), 13,078 participants were randomized to receive 4 g of EPA+DHA per day or placebo (corn oil) (34). Cardiovascular events were similar between the treatment groups (HR: 0.99; 95% CI: 0.90–1.09; *P* = 0.84) even though there were greater reductions in triglycerides (−19.0 vs. −0.9%) and non–HDL-C (−6.1 vs. −1.1%) in those who received EPA+DHA compared with corn oil. n-3 PUFA supplements were associated with more AF and gastrointestinal adverse events compared with placebo.

The OMEMI trial is a Norwegian investigator-initiated RCT in 1,014 elderly patients (mean ± SD age was 75 ± 3.6 years) with triglycerides of 1.3 mmol/L (35). As in the STRENGTH trial there was no benefit of EPA+DHA given in the dose of 1.8 g per day. The primary endpoint occurred in 108 (21.4%) patients on n-3 PUFA versus 102 (20.0%) on placebo (HR: 1.08; 95% CI: 0.82–1.41; *P* = 0.60).

In 2022, an SR on 15 RCTs found reduced incidence of major cardiovascular events in the n-3 fatty acid group (RR: 0.95, 95% CI: 0.91–0.99, *P* = 0.026), myocardial infarction (RR: 0.90, 95% CI: 0.83–0.98; *P* = 0.021), and cardiovascular death (RR: 0.94, 95% CI: 0.88–0.99; *P* = 0.028) compared with the control group (48).

## Summary

Studies indicate that eating fish, particularly fish rich in n-3 PUFA, at least once a week, is associated with a lower risk of CVD. However, several meta-analyses show no benefits of supplementary fish oils on CVD. Recent RCTs have generated new important and somewhat conflicting results. Studies on 4 g daily of EPA showed beneficial effect, but mixed EPA+DHA formulations have not shown any clear incremental clinical benefit on top of statin therapy. Notably, the 2021 ESC Guidelines on CVD prevention in clinical practice downregulated the documentation on 4 g EPA from class IIa to class IIb in high-risk patients. More studies are needed on the effectiveness of 20- and 22-carbon atoms n-3 PUFA in CVD.

### Saturated fatty acids

We identified eight qSRs on the effect of SFA on CVD. Here we summarize some of the most recent ones.

Hooper (49) included 15 RCTs (16 comparisons, ~59,000 participants). The included trials suggested that reducing dietary SFA reduced the risk of combined cardiovascular events by 17% (RR: 0.83 [0.70–0.98], *I*^2^ = 67%, 12 RCTs, GRADE moderate-quality evidence). Greater reductions in SFA (reflected in greater reductions in serum cholesterol) resulted in greater reductions in risk of CVD events. In primary prevention trials and secondary prevention trials, 56 and 53 people needed to reduce their SFA intake for ~4 years for one person to avoid experiencing a CVD event, respectively. Subgrouping did not suggest significant differences between replacement of SFA with PUFA or carbohydrate, and data on replacement with MUFA and protein were very limited. There was little or no effect of reducing SFA on cardiovascular mortality (RR: 0.95 [0.80–1.12], *I*^2^ = 30%, 10 RCTs), with GRADE moderate-quality evidence. The lack of effect on cardiovascular mortality remained, regardless of what was substituted for SFA. There was little or no effect of reducing SFA on nonfatal myocardial infarction (RR: 0.97 [0.87–1.07], *I*^2^ = 0%, seven RCTs) or CHD mortality (RR: 0.97 [0.82–1.16], *I*^2^ = 28%, eight RCTs), both with low-quality evidence. In subgroup analyses, benefits for nonfatal myocardial infarction were indicated when SFA was replaced with PUFA (RR: 0.80 [0.63–1.03], *I*^2^ = 0%, five RCTs), but not other dietary replacements. For CHD mortality, there was no suggestion of an effect regardless of what replaced SFA. Effects on total (fatal or nonfatal) myocardial infarction, stroke, and CHD events (fatal or nonfatal) were all unclear as the evidence was of very low quality. There was little or no effect on HDL-C or serum triglycerides. Finally, there was no evidence of harmful effects of reducing SFA intakes. In summary, the authors suggest that reducing SFA intake for at least 2 years causes a potentially important reduction in combined cardiovascular events. Replacing the energy from SFA with PUFA or carbohydrate appears to be a useful strategy. Greater reduction in SFA caused greater reductions in cardiovascular events.

The 2020 Dietary Guidelines Advisory Committee from the United States Department of Agriculture (27) reviewed 228 articles in total (37 articles on children, 191 articles on adults). They included several different types of studies, RCTs, non-RCTs, prospective cohort studies, retrospective cohort studies, and nested case-control studies. They did not use case-control studies, cross-sectional studies, and uncontrolled before-and-after studies, as well as meta-analyses and reviews. They found strong evidence that replacing SFA with PUFA in adults reduces the risk of CHD events and CVD mortality. However, evidence is insufficient to determine whether replacing SFA with different types of carbohydrates (e.g. complex, simple) in adults affects the risk of CVD. As a result of limited available evidence, it is unclear if MUFAs reduce CVD risk. Furthermore, it cannot be determined whether replacing SFA with PUFA in adults affects the risk of stroke or heart failure because of insufficient evidence. Furthermore, insufficient evidence was available to quantify an independent relationship between dietary cholesterol intake in adults and blood lipids as well as overall risk of CVD. Regarding blood lipids, they found strong and consistent evidence from RCTs that replacing SFA with unsaturated fats, especially PUFA, in both adults and children significantly reduces total- and LDL-C. Replacing SFA with carbohydrates (sources not defined) also reduces total- and LDL-C in adults, but significantly increases triglycerides and reduces HDL-C.

Using observational studies, de Souza et al. (50) reported that SFA intake was not associated with CVD mortality (0.97 [0.84–1.12]), total CHD (1.06 [0.95–1.17]), or ischemic stroke (1.02 [0.90–1.15]). The certainty of associations between saturated fat and all outcomes was ‘very low’.

Reynolds et al. performed an SR and meta-analysis of 112 publications (3,696,568 participants) related to SFA. CHD incidence was reduced with a 5 E% replacement with PUFA (RR: 0.89 [0.81–0.98]), plant MUFAs (RR: 0.83 [0.69–1.00]), and slowly digested carbohydrates (RR: 0.94 [0.89–0.99]). For CHD, there was no reduction in risk when replacement was with rapidly digested carbohydrates (RR: 1.08 [0.99–1.17]) or animal MUFAs (RR: 1.06 [0.80–1.41]) and replacing SFA with animal protein was associated with increased risk (RR: 1.31 [1.14–1.50]). The risk of CVD was reduced with a 5 E% replacement with PUFA (RR: 0.90 [0.81–1.00]) and plant MUFAs (RR: 0.90 [0.84–0.96]), but not with total MUFAs (RR: 0.94 [0.87–1.02]) or carbohydrates (RR: 0.98 [0.90–1.07]). Higher (compared to lower) intake of SFA was not associated with risk of ischemic stroke (RR: 0.96 [0.87–1.12]).

Wolfram et al. (31) report that there is probable evidence that the substitution of SFA with PUFA lowers risk of CHD and there is convincing evidence that it lowers the plasma concentration of total- and LDL-C, in line with conclusions from Schwab et al. (26).

### Results from non-qualified systematic reviews

An SR from 2018 (51) investigated the differential effects of medium- and long-chain SFA on blood lipid profile. Diets enriched with medium-chain SFAs led to significantly higher HDL-C concentrations than diets enriched with long-chain SFAs (0.11 mmol/L [0.07–0.15 mmol/L]) with no effect on triglyceride, LDL-C, and total cholesterol concentrations.

An SR and meta-analysis from 2021 (52) of prospective studies investigated the association between circulating or adipose tissue levels (highest vs. lowest) of the proposed dairy fat biomarkers 15:0, 17:0 and t16:1n-7 and CVD. Higher levels of 15:0 was inversely associated with total CVD (RR: 0.88 [0.78–0.99], *I*^2^ = 58.6%, *n* = 17 studies). In subgroup analyses, no associations were found for CVD incidence or mortality, CHD incidence or mortality, stroke incidence or mortality, or heart failure (*n* = 1–9 studies for each). Higher levels of 17:0 was inversely associated with total CVD (RR: 0.86 [0.79–0.93], *I*^2^ = 0%, *n* = 12 studies). In subgroup analyses, higher levels of 17:0 was inversely associated with CHD incidence (RR: 0.86 [0.78–0.96]), stroke incidence (RR: 0.87 [0.77–0.98]), and stroke mortality (RR: 0.63 [0.43–0.93]) but not CVD incidence or mortality, CHD mortality or heart failure (*n* = 1–6 studies for each). Levels of t16:1n-7 were not associated with total CVD (RR: 1.01 [0.91–1.12], *I*^2^ = 0%, *n* = 6 studies). Subgroup analyses indicated no associations to CVD incidence or mortality, CHD incidence or mortality, stroke incidence or mortality or heart failure (*n* = 1–4 studies for each).

### Trans fatty acids

There were three qSRs reporting on TFA and risk of CVD (50, 53, 54), one qSR reporting on risk markers (31) and the other reporting on SFA and TFA in children and adolescents (55).

Brouwer et al. (53) only included RCTs with at least one intervention group with either increased industrial TFA or ruminant TFA intake. The study showed that replacement of TFA from any source by cis-PUFA consistently lowers total cholesterol, LDL-C, and ApoB for all TFAs studied. Replacement of industrial TFA by MUFAs, PUFAs, or carbohydrates led to increased levels of HDL-C, decreased levels of total cholesterol and LDL-C with replacement by PUFA showing the strongest effects. Only replacement with PUFA showed a significant effect on reducing triglycerides. Outcomes for ruminant TFA were in the same direction as for industrial TFA. Regarding TFA, no trials were identified meeting the inclusion criteria in children.

de Souza et al. (50) used observational studies reporting associations of SFA and/or TFA (total, industrially manufactured, or from ruminant animals) with all-cause mortality, CHD/CVD mortality, total CHD, ischemic stroke, or T2D. Total TFA intake was associated with all-cause mortality (1.34 [1.16–1.56]), CHD mortality (1.28 [1.09–1.50]), and total CHD (1.21 [1.10–1.33]) but not ischemic stroke (1.07 [0.88–1.28]) or T2D (1.10 [0.95–1.27]). Industrial, but not ruminant, TFAs were associated with CHD mortality (1.18 [1.04–1.33] vs. 1.01 [0.71–1.43]) and CHD (1.42 [1.05–1.92] vs. 0.93 [0.73–1.18]). Ruminant trans-palmitoleic acid was inversely associated with T2D (0.58 [0.46–0.74]). The certainty of associations of trans fat with CHD outcomes was ‘moderate’ and ‘very low’ to ‘low’ for other associations.

Reynolds et al. (54) reported that higher (compared to lower) dietary intakes of TFA were associated with increased risk of CHD (RR: 1.17 [1.09–1.27]) and CVD (RR: 1.14 [1.04–1.25]), but not ischemic stroke. TFA intake >1 E% was associated with increased risk of CHD (RR: 1.14 [1.04–1.25]) and CVD (RR: 1.20 [1.08–1.33]) compared to intake <1% of total energy. A 2 E% replacement of TFA with plant (but not animal) MUFAs was associated with reduced CHD (RR: 0.80 [0.70–0.92]) but not CVD. Higher (compared to lower) intake of industrially produced TFA was associated with increased risk of CHD (RR: 1.28 [1.10–1.50]) but higher intake of ruminant-derived TFA was not associated with CHD (RR: 0.95 [0.75–1.20]). Tissue levels of TFA were not associated with risk of CHD or CVD.

Te Morenga et al. (55) examined the evidence for health effects associated with reducing SFA and TFA intake in free-living children, adolescents, and young adults between 2 and 19 years of age (RCTs) and prospective cohort studies. Compared with control diets, there was a highly statistically significant effect of reduced SFA intake on total cholesterol (MD −0.16 mmol/L [−0.25 to −0.07]), LDL-C (MD −0.13 mmol/L [−0.22 to −0.03]) and diastolic blood pressure (MD −1.45 mmol/L [−2.34 to −0.56]). There were no significant effects on any other risk factors and no evidence of adverse effects. Regarding TFA, no trials were identified meeting the inclusion criteria.

The study of Wolfram (31) reported that dietary TFA increases the total- and LDL-C concentration in plasma as well as reduction of the HDL-C concentration in plasma and increase in the triglycerides. This evidence was classified as convincing. Furthermore, they report that the ratio of total cholesterol to HDL-C increases but found insufficient evidence to conclude that TFAs change the ratio of LDL-C to HDL-C. They also concluded that evidence is insufficient to claim a different effect of industrial and natural TFA from ruminants. Regarding TFA of the specific type of conjugated linoleic acid (CLA), they report that evidence of effects of CLA on the total- and LDL-C concentration in plasma is insufficient. This applies to evidence for an influence of CLA on the HDL-C and for a change of the triglycerides as well as effects on the ratio of total- to LDL-C and of LDL-C to HDL-C. Wolfram et al. (31) conclude that there is convincing evidence that a high intake of TFA increases risk of dyslipidemia.

### Results from non-qualified systematic reviews

An SR from 2011 (56) assessed the association between intake of TFA and the risk of CHD, with a specific emphasis on distinguishing between TFA of industrial and ruminant origin. The authors suggest that industrial TFA may be positively related to CHD, whereas ruminant TFA is not, but the limited number of available studies prohibits any firm conclusions concerning whether the source of TFA is important. An SR from 2020 (57) comparing the effect of ruminant and industrial TFA on cardiometabolic risk markers identified six trials reporting effects on blood lipids. Although results were heterogeneous, the authors conclude that ruminant TFA may increase total cholesterol, LDL-C, and triglycerides more than industrial TFA, whereas industrial TFA may lower HDL-C more than ruminant TFA. The authors noted that ruminant TFA had a different effect in women than men in one intervention study. Among women, both HDL-C and LDL-C were higher after intake of 5 E% ruminant TFA compared with equivalent intakes of industrial TFA, whereas only minor differences were observed in men (57).

### Effects of saturated and trans fatty acids in children and adolescents

Te Morenga et al. (55) reviewed RCTs with dietary interventions aiming to reduce SFA or TFA intakes and a control group in addition to cohort studies reporting the effects of SFA or TFA exposures, on outcomes including blood lipids, measures of growth, blood pressure, insulin resistance, and potential adverse effects. Minimum duration was 13 days for RCTs and 1 year for cohort studies. Trials of weight loss or confounded by additional medical or lifestyle interventions were excluded. Compared with control diets, there was a highly statistically significant effect of reduced SFA intake on total cholesterol (MD −0.16 mmol/L [−0.25 to −0.07]), LDL-C (MD −0.13 mmol/L [−0.22 to −0.03]), and diastolic blood pressure (MD −1.45 mmol/L [−2.34 to −0.56]). There were no significant effects on any other risk factors and no evidence of adverse effects. Advice to reduce SFA intake of children results in a significant reduction in total- and LDL-C levels as well as diastolic blood pressure without evidence of adverse effects on growth and development. The authors conclude that dietary guidelines for children and adolescents should continue to recommend diets low in SFA.

### Dietary cholesterol and plasma lipoproteins

The 2020 DGAC (27) systematically reviewed the current evidence. They noted that dietary cholesterol is commonly found in foods that also contain SFA, making it difficult to assess dietary cholesterol independently. They reviewed the evidence for children and adults separately and reported results from RCT and prospective cohort studies separately.

For children, they report that results from RCTs that modified SFA, PUFA, and dietary cholesterol intake were predominantly consistent with one another, showing primarily beneficial effects throughout childhood for total cholesterol and LDL-C. The RCTs modified child fat intake either through dietary counseling that focused primarily on reducing SFA and dietary cholesterol intake, with additional encouragement to increase PUFA intake, or through provision of food products (i.e. eggs, extra virgin olive oil, or oily fish) that differed in types of fat including SFA, MUFA, PUFA, and/or dietary cholesterol. Evidence from RCTs predominantly indicated that consuming less SFA and dietary cholesterol resulted in lower blood total cholesterol and LDL-C throughout childhood, particularly in boys. Most studies were conducted in the United States, the United Kingdom, and Scandinavia. The majority of studies included children with normal blood lipids at baseline, although some specifically recruited children with elevated or higher than average blood lipid levels. The evidence from prospective cohort studies was less consistent than from the RCTs. The majority of studies assessed blood lipids during childhood; few assessed intakes of types of fat during childhood and blood lipids into early adulthood. Although risk of publication bias is always of potential concern, both small and large studies were included in this review, reporting both null and statistically significant results. Therefore, risk of publication bias is likely low across this body of evidence. Overall, the 2020 DGAC determined the evidence demonstrates that diets lower in SFA and cholesterol during childhood result in lower levels of total- and LDL-C throughout childhood, particularly in boys.

In adults, nine articles (four from parallel design RCTs and five from crossover design RCTs) primarily assessed the relationship between dietary cholesterol intake and blood lipids. Of these, most achieved differences in dietary cholesterol intake by providing the intervention arm with meals or beverages that contained whole eggs or egg yolks and the comparator arm with dietary cholesterol-free alternatives such as egg substitutes, egg whites, or carbohydrate-based breakfast foods (e.g. oatmeal). Across the body of evidence, higher dietary cholesterol intake, compared to lower dietary cholesterol intake, had either null effects on blood lipids (7 articles) or resulted in higher levels of total blood cholesterol (3 articles), LDL-C (2 articles), HDL-C (2 articles), and/or triglycerides (1 article). They summarized that predominantly null effects were reported for dietary cholesterol. However, among the few articles that found significant results, higher intake of dietary cholesterol, compared to lower intake, significantly increased or resulted in higher levels of total-, LDL-C, and HDL-C. In several articles, it was not possible to isolate the independent effect of dietary cholesterol on blood lipids because of simultaneous changes in the total amount of fat or proportion of different types of fatty acids in the study diet. They concluded that insufficient evidence is available from RCTs to quantify an independent relationship between dietary cholesterol intake in adults and overall risk of CVD.

Eleven articles (10 articles from eight prospective cohort studies and one article from a nested case-control study) assessed the relationship between dietary cholesterol intake and CVD endpoint outcomes. Of these, five examined CVD, inclusive of multiple types of outcomes, four examined CHD or four examined stroke, and two examined heart failure. For CVD, results were inconsistent. One article, which included multiple large and well-characterized cohorts and used validated diet assessment methods, reported statistically significant associations between higher intake of dietary cholesterol and increased risk of CVD and CVD mortality, whereas other articles reported statistically significant associations with decreased risk of CVD or reported null associations. Few articles, with inconsistent results, assessed the independent relationship between dietary cholesterol intake and CVD endpoint outcomes, thereby further confounding meaningful conclusions. The 2020 DGAC could therefore not draw a conclusion about the relationship between dietary cholesterol intake and blood lipids or risk of CVD due to a small number of studies and inconsistent results.

Due to the co-occurrence of dietary cholesterol in foods that are also high in SFA and the relatively low dietary cholesterol intake in several articles, it was difficult to disentangle the independent associations between dietary cholesterol and CVD endpoint outcomes in this body of evidence.

### Concluding remarks

Overall, the evidence presented earlier strengthens the current advice to reduce the consumption of saturated fat in the Nordic and Baltic countries. Replacing saturated fat with unsaturated fat provides the largest reduction in LDL-C and can reduce CVD caused by atherosclerosis. Both SFA and TFA should be replaced by PUFAs, plant MUFAs, and slowly digested carbohydrates.

#### Hypertension and blood pressure

In the qSR by Hooper et al. from 2020 investigating the effects of total fat intake on body fatness (16), it was found that diets with reduced fat content decreased both systolic blood pressure (MD −0.75 mm Hg [−1.42 to −0.07]) and diastolic blood pressure (MD −0.52 mm Hg [−0.95 to −0.09]) compared to diets higher in total fat content.

In the qSR by Hooper et al. from 2020 investigating the effects of reducing SFA intake for prevention of CVD (49), no effect on systolic (−0.19 mm Hg [−1.36 to 0.97]) or diastolic (−0.36 mm Hg [−1.03 to 0.32]) blood pressure was found for diets lower compared to higher in SFA.

The qSR by Wolfram et al. from 2015 (31) found insufficient evidence on the intake of MUFA and total PUFA in relation to blood pressure or risk of hypertension. The authors found probable evidence for a lack of association between n-6 PUFA and blood pressure and risk of hypertension. However, there is probable evidence that long-chain n-3 PUFAs have a blood pressure–reducing effect, but that this requires high doses inconvenient to consume with conventional foods.

#### Results from non-qualified systematic reviews

An SR and meta-analysis from 2011 (58) comparing high-MUFA (>12 E%) with low-MUFA (<12 E%) diets on cardiometabolic risk markers identified nine randomized trials reporting effects on blood pressure. Compared to low-MUFA diets, high-MUFA diets reduced both systolic blood pressure (−2.26 mm Hg [−4.28 to −0.25], *I*^2^ = 50%) and diastolic blood pressure (−1.15 mm Hg [−1.96 to −0.34], *I*^2^ = 3%).

An SR from 2020 (57) comparing the effect of ruminant and industrial TFA on cardiometabolic risk markers identified two trials reporting effects on blood pressure, with no differential effects of ruminant and industrial TFA on systolic or diastolic blood pressure.

### Concluding remarks

A reduced total fat content has beneficial effect on blood pressure and compared to diets higher in total fat content according to one qSR from 2020. Regarding effects of particular fatty acids, one qSR found a blood pressure–lowering effect of omega-3 fatty acids. No association was found between SFA and blood pressure in two qSRs. One non-qSR found that MUFA had a blood pressure–lowering effect. Overall, a reduced fat intake in the population could have a beneficial effect on blood pressure.

#### Glucose metabolism and type 2 diabetes

In the qSR by Hooper from 2020 investigating the effect of reduction in SFA intake for CVD prevention (49), no effect on diabetes diagnoses was found for diets lower compared to higher in SFA (RR: 0.96 [0.90–1.02]); however, this was based on a single trial (Women’s Health Initiative).

Using observational studies, de Souza et al. (50) reported that SFA intake (highest vs. lowest) was not associated with T2D (RR: 0.95 [0.88–1.03], *I*^2^ = 0%, based on eight studies). Intake of total TFA was not associated with T2D (RR: 1.10 [0.95–1.27], *I*^2^ = 66%, based on six studies) but intake of ruminant TFA (assessed by levels of t16:1n-7) was inversely associated with T2D (RR: 0.58 [0.46–0.74], *I*^2^ = 30%, based on five studies).

Using observational data, Reynolds et al. (54) reported that higher (compared to lower) intake of SFA was not associated with risk of T2D (RR: 1.02 [0.95–1.10]). Similarly, intakes of <10 E% from SFA compared to >10 E% was not associated with T2D (RR: 1.01 [0.82–1.23]). A 5 E% replacement of SFA with carbohydrates (RR: 1.05 [0.99–1.11]) or PUFA (RR: 0.96 [0.85–1.08]) was not associated with T2D. Higher tissue levels of 15:0 (RR: 0.79 [0.68–0.93]) and 17:0 (RR: 0.66 [0.52–0.84]) were associated with reduced risk of T2D. Higher (compared to lower) intake of total TFA was not associated with risk of T2D (RR: 1.05 [0.95–1.16]). Tissue levels of total TFA were not associated with T2D (RR: 0.77 [0.40–1.48]). For individual isomers, tissue levels of t16:1n-7 (RR: 1.25 [1.06–1.48]) and t18:1n-9 (RR: 1.20 [1.04–1.38]) were positively associated with T2D whereas t16:1n-9 was not associated (RR: 1.30 [0.82–2.08]).

Brown et al. (59) identified 83 RCTs of at least 24 weeks’ duration assessing effects of increasing ALA, long-chain n-3 (the majority of studies), n-6, or total PUFA on diabetes diagnosis and glucose metabolism. Long-chain n-3 PUFA had little or no effect on diabetes diagnosis (RR: 1.00 [0.85–1.17]), HbA1c (−0.02 [−0.07 to 0.04]), plasma glucose (0.04 [0.02–0.07]), fasting insulin (1.02 [−4.34 to 6.37]), or HOMA-IR (0.06 [−0.21 to 0.33]). Effects of ALA, n-6, and total PUFA on diabetes diagnosis were unclear (very low-quality evidence) but little or no effect on measures of glucose metabolism was observed. Finally, no evidence was found that the n-3:n-6 PUFA ratio is important for diabetes or glucose metabolism. The authors conclude that increasing n-3, n-6, or total PUFA has little or no effect on prevention and treatment of T2D mellitus.

#### Results from non-qualified systematic reviews and biomarker studies

In an SR from 2016 (60), Imamura et al. investigated the effects of SFA, PUFA, MUFA, and carbohydrates on glucose-insulin homeostasis, including 102 RCTs. The authors found that replacing SFA with PUFA decreased fasting glucose (−0.04 [−0.07 to −0.01]), and that isocaloric replacement of 5 E% from either SFA or carbohydrate with 5 E% from either MUFA or PUFA lowered HbA1c. These results differ from those of Brown et al. described earlier. This may be due to the fact that Brown et al. primarily assessed n-3 PUFA and/or differences in intervention duration (median feeding duration 28 days vs. at least 24 weeks).

An individual participant-level pooling project of 20 prospective cohort studies investigated the association between n-3 PUFA biomarkers in plasma/tissue and incidence of T2D (61). The authors found that EPA (HR: 0.92 [0.87−0.96]), DPA (0.79 [0.73−0.85]), DHA (0.82 [0.76−0.89]), and their sum (0.81 [0.75−0.88]), but not ALA (0.97 [0.92−1.02]), were inversely associated with incident T2D during follow-up ranging from 2.5 to 21.2 years. Findings were robust across subgroups and sensitivity analyses and upon extensive adjustment for sociodemographic factors, lifestyle habits, medical diagnoses, and adiposity.

An SR from 2020, based on 23 prospective cohort studies, investigated the association between dietary fats and incidence of T2D (62). In linear dose-response (per 10 g/day) meta-analyses, neither total fat (RR: 1.00 [0.96−1.05], *I*^2^ = 78%, *n* = 8 cohorts) nor animal fat (RR: 1.03 [1.00−1.06], *I*^2^ = 0%, *n* = 5 cohorts) or vegetable fat (RR: 0.93 [0.82−1.05], *I*^2^ = 91%, *n* = 5 cohorts) was associated with incidence of T2D. Similarly, intakes of SFA (RR: 0.97 [0.92−1.07], *I*^2^ = 34%, *n* = 11 cohorts), MUFA (RR: 1.03 [0.99−1.08], *I*^2^ = 0%, *n* = 10 cohorts), PUFA (RR: 1.03 [0.89−1.20], *I*^2^ = 76%, *n* = 8 cohorts), and LA (RR: 0.99 [0.96−1.01], *I*^2^ = 65%, *n* = 6 cohorts) were not associated with incidence of T2D. Similar lack of associations was observed for n-3 PUFA, EPA, DHA, ALA, and TFA. However, in non-linear analyses, vegetable fat was associated with decreased risk of T2D incidence up to 13 g/day (RR: 0.81 [0.76−0.88]), after which the curve plateaued.

Another SR and meta-analysis from 2020 investigated the association between linoleic acid, both self-reported and biomarkers, and risk of T2D in 31 prospective cohorts (63). The authors found that high, compared to low, intake of LA was associated with a 6% lower risk of T2D (RR: 0.94 [0.90−0.99]), and in dose-response analysis, each 5 E% increment of LA was associated with a 10% lower risk. In accordance, every SD increment in LA in blood/adipose tissue was associated with 15% lower risk of T2D (RR: 0.85 [0.80−0.90], *I*^2^ = 66.2%).

An individual-level pooled analysis from 2018 investigated if odd-chain fatty acids 15:0 and 17:0 and trans-palmitoleic acid (t16:1n-7), potentially partly reflecting intake of dairy fat, were associated with incident T2D in 16 prospective cohort studies (64). They found that higher levels of 15:0, 17:0, and t16:1n-7 in circulation or adipose tissue were associated with lower incidence of T2D. In the most adjusted model, the HR (95% CI) for incident T2D per cohort-specific 10th to 90th percentile range of 15:0 was 0.80 (0.73−0.87); of 17:0, 0.65 (0.59−0.72); of t16:1n7, 0.82 (0.70−0.96); and of their sum, 0.71 (0.63−0.79).

An SR and meta-analysis from 2012 (65) identified seven randomized trials comparing higher and lower intakes of TFA. There was no differential effect on either glucose (0.08 [−0.14 to 0.30], *I*^2^ = 0%) or insulin (−0.02 [−0.23 to 0.20], *I*^2^ = 0%) levels. An individual-level pooled analysis of prospective cohorts from 2022 investigated circulating levels of TFA and risk of T2D (66). In 12 cohorts with a mean follow-up of 13.5 years, higher levels of trans/trans-18:2, cis/trans-18:2, and trans/cis-18:2 were not associated with incidence of T2D, whereas higher levels of trans-16:1n-9, total trans-18:1, and total trans-18:2 were inversely associated.

An SR from 2020 (57) comparing the effect of ruminant and industrial TFA on cardiometabolic risk markers identified three trials reporting on glucose, insulin, and insulin sensitivity (HOMA). There were no differential effects of ruminant and industrial TFA on any of the outcomes.

Regarding type 1 diabetes, only one SR was identified (67). This study was based on three prospective cohort studies and four case-control studies on PUFA intake during pregnancy or during early life in children. Supplementation with n-3 PUFA had no effect on preclinical type 1 diabetes overall (OR: 0.98 [0.85−1.13], *I*^2^ = 36.1%) or during pregnancy (OR: 1.00 [0.91−1.10]) but indicated protective effects during early life (OR: 0.45 [0.21−0.96]). Similarly, n-3 PUFA had no effect on clinical type 1 diabetes (RR: 0.87 [0.71−1.08], *I*^2^ = 64.7%), with similar effects during pregnancy (RR: 1.00 [0.83−1.20]) and early life (RR: 0.72 [0.45−1.16]). Finally, supplementation with n-6 PUFA had no effect on preclinical (RR: 1.07 [0.97−1.17], *I*^2^ = 0%) or clinical type 1 diabetes (RR: 1.05 [0.92−1.20], *I*^2^ = 0%).

### Concluding remarks

Data from four qSRs have all shown that specific fatty acids do not affect the risk of T2D, except that one qSR from 2015 found that intake of ruminant TFA (assessed as t16:1n-7) was inversely associated with T2D. The latter is supported by biomarker studies, showing inverse associations between levels of t16:1n-7, as well as 15:0 and 17:0 (potentially partly reflecting intake of dairy fat), with incidence of T2D. Biomarker studies also show protective associations for both LA and long-chain n-3 PUFA for incidence of T2D. Taken together, current evidence is inconsistent and somewhat difficult to reconcile but indicates that the risk of T2D may not be much affected by any specific fatty acids, although protective associations are shown for multiple fatty acids.

#### Cancers

Two qSRs, Schwab (26) and Wolfram (31), were available that investigated the association between fat intake and cancer. Taken together, there are no strong associations between intake of total fat or the quality of fat and individual cancers, but the evidence is inconclusive. In addition to these two qSRs, multiple, more recent, SRs were identified through the literature review. The evidence from these is summarized below for individual cancer types. The majority of identified SRs had included studies with both cohort- and case-control designs; however, the evidence summarized below is primarily based on findings from prospective studies as this was available for all outcomes.

#### Any cancer

*n-6 and n-3 PUFAs.* A meta-analysis from 2020 of prospective studies investigated the association between dietary intake and blood levels of n-6 PUFA and risk of any cancer (68). Based on 46 studies including almost 100,000 cancer cases, there was no association between self-reported intake of n-6 PUFA (highest vs. lowest) and any cancer (RR: 1.02 [0.99–1.05] with moderate heterogeneity [*I*^2^ = 44.3%]). Similarly, there were no significant associations in dose-response analysis or in subgroups on cancer site, type of n-6 PUFA, or sex. However, based on 29 studies including almost 14,000 cancer cases investigating blood levels of n-6 PUFA, an inverse association (highest vs. lowest) was observed (RR: 0.92 [0.86–0.98] without significant heterogeneity [*I*^2^ = 13.8%]). In subgroup analyses, results appear to be driven by breast cancer (RR: 0.87 [0.77−0.98], *n* = 11) and LA (RR: 0.91 [0.82−1.00], *n* = 28) and overall associations were slightly stronger in women (RR: 0.88 [0.79−0.97], *n* = 13) than in men (RR: 0.92 [0.83−1.02], *n* = 13). The multivariable adjusted dose-response analysis showed that a 5% increase in blood levels of n-6 PUFA was associated with a 2% lower risk of any cancer (RR: 0.98 [0.97−0.99]).

An individual-level pooled analysis from 2021 studied circulating levels of n-3 PUFA and risk of cancer mortality (38). Among 15 prospective cohorts with a median of 16 years of follow-up, higher compared to lower levels of EPA (HR: 0.82 [0.74–0.91]), DPA (HR: 0.79 [0.70–0.90]), and DHA (HR: 0.86 [0.78–0.95]) were associated with lower risk for cancer mortality.

*Total PUFA.* In the qSR by Hooper from 2020 investigating the effect of reduction in SFA intake for CVD prevention (49), no effect on cancer diagnoses (RR: 0.94 [0.83–1.07], based on four trials) or cancer deaths (RR: 1.00 [0.61–1.64], based on five trials) was found for diets lower compared to higher in SFA.

An SR and meta-analysis from 2021 (40), based on 19 prospective cohort studies including 1,013,273 participants and 195,515 deaths, found that a 5 E% increase in PUFA was associated with 4% (RR: 0.96 [0.94–0.99]) lower mortality from cancer. The risk of cancer mortality increased by 4% for every 5% increase in energy from SFA (RR: 1.04 [1.02–1.06]).

*MUFA.* An SR and dose-response meta-analysis from 2021 (43), based on 10 effect sizes from six prospective cohort studies (including a total of 64,448 deaths), found no association between dietary intake of MUFA (highest vs. lowest) and cancer mortality (RR: 0.99 [0.96–1.03], *I*^2^ = 13%). Similarly, a 5 E% incremental intake from MUFA was not associated with risk of cancer mortality (RR: 0.99 [0.97–1.01]).

#### Prostate cancer

*Long-chain n-3 PUFA.* In a meta-analyses from 2015 (69), no association between long-chain n-3 PUFA (EPA, DPA, DHA) and incidence of prostate cancer was observed. Results were similar when based on self-reported dietary intakes (*n* = 13 studies, high vs. low intake) and biomarkers (*n* = 10 studies). In sub-analyses, results for self-reported dietary intake were similar when stratified by follow-up duration (more or less than 10 years) but indicated harm in biomarker studies where follow-up was less than 10 years (*n* = 6 studies, RR: 1.12 [0.99–1.27]) but no indication with longer follow-up (*n* = 4 studies, RR: 0.93 [0.71–1.22]). All studies were conducted in North America, Europe, and Australia. Similarly, an SR from 2017 (70) based on 19 analyses reached the conclusion that the evidence is insufficient to suggest a relationship between intake of long-chain n-3 PUFA and incident prostate cancer. In contrast, a dose-response meta-analysis of prospective cohort studies from 2015 (71) observed different associations for individual n-3 PUFA. Dietary ALA was very marginally inversely associated (RR: 0.99 [0.98–1.00] per 0.5 g/day increase; *n* = 5 studies) whereas dietary DHA was positively associated and dietary EPA was not associated. Furthermore, levels of DHA in blood were marginally positively associated (RR: 1.02 [1.00–1.05] per 1% increase; *n* = 9 studies) whereas levels of EPA (RR: 1.01 [0.99–1.04] per 0.5% increase; *n* = 9 studies) and ALA (RR: 1.00 [0.98–1.03] per 0.1% increase; *n* = 9 studies) were not and levels of DPA was inversely associated (RR: 0.97 [0.94–1.00] per 0.2% increase; *n* = 5 studies). Finally, the latest meta-analysis from 2021 (72) observed no association (highest vs. lowest) for ALA (RR: 0.99 [0.86–1.15], *n* = 7) but indications for increased risk for total n-3 PUFA (RR: 1.04 [1.00–1.08], *n* = 3).

*n-6 PUFA.* A meta-analysis from 2020 (68) based on 10 prospective studies observed no association between self-reported intake of n-6 PUFA and risk of prostate cancer (RR: 1.02 [0.99–1.06]). Similarly, blood levels of n-6 PUFA were not associated with risk of prostate cancer (RR: 0.94 [0.84–1.05], *n* = 10 cohorts). A lack of association for self-reported intake of n-6 PUFA was also observed in the latest meta-analysis from 2021 (72) (highest vs. lowest, RR: 0.97 [0.82–1.16], *n* = 6).

*Total fat, SFA, MUFA, PUFA.* In a dose-response meta-analysis of prospective cohorts from 2015 (73), risk of prostate cancer was not associated with intake of total fat (RR: 1.00 [0.99–1.01] per 28 g increment; *n* = 13 studies), SFA (RR: 1.00 [1.00–1.00] per 28 g increment; *n* = 9 studies), MUFA (RR: 1.00 [0.95–1.04] per 28 g increment; *n* = 8 studies), PUFA (RR: 0.99 [0.95–1.03] per 28 g increment; *n* = 7 studies), or total unsaturated fat (RR: 0.99 [0.96–1.02] per 28 g increment; *n* = 10 studies). Results were consistent in subgroup analyses adjusted for BMI.

#### Skin cancer

Based on self-reported intake data from three individual cohorts, there was no association between consumption of n-3 PUFA and incidence of basal cell carcinoma (BCC) or squamous cell carcinoma (SCC) (74). Furthermore, based on self-reported data from a few individual cohorts, there was no association between consumption of total fat, SFA, MUFA, or PUFA and incidence of BCC, SCC, or cutaneous malignant melanoma (CMM) (75).

A meta-analysis from 2020 (68) based on three prospective studies observed no association between self-reported intake of n-6 PUFA and risk of skin cancer (RR: 1.02 [0.80–1.29]).

#### Pancreatic cancer

In a meta-analysis based on six individual cohorts, there was no association (RR: 1.05 [0.85–1.29]) between self-reported intake of total fat and risk of pancreatic cancer (76). Similarly, in a meta-analysis of cohort studies, there was no association between self-reported consumption of SFA (*n* = 6 cohorts), MUFA (*n* = 5 cohorts), or PUFA (*n* = 6 cohorts) and risk of pancreatic cancer (77). A meta-analysis from 2020 (68) based on four prospective cohorts observed no association between self-reported intake of n-6 PUFA and risk of pancreatic cancer (RR: 0.99 [0.86–1.14]).

#### Ovarian cancer

In a meta-analysis (78) from 2016, based on six cohorts, there was no association between self-reported intake of total fat and risk of ovarian cancer (RR: 1.10 [0.97–1.24]). Similar null results were observed for SFA, MUFA, PUFA, trans fat, dairy fat, plant fat, and animal fat (based on 2–5 cohorts each). When the majority of these cohorts (and some additional) were combined with case-control studies in a dose-response meta-analysis from 2019 (79), significant but marginal associations were observed for total fat (RR: 1.02 [1.01–1.02] per 10 g increment) and trans fat (RR: 1.02 [1.01–1.03] per 0.5 g increment), whereas SFA and MUFA were nonlinearly associated (risk increased with intakes over ~25 g/day for both SFA and MUFA). Unfortunately, prospective studies were not reported separately from case-control studies and this result should thus be interpreted cautiously as the prospective studies on their own did not indicate clear associations.

#### Non-Hodgkin lymphoma

A meta-analysis from 2017 (80) based on two cohorts of American women observed a positive association between self-reported intake (highest vs. lowest) of total fat and risk of non-Hodgkin lymphoma (RR: 1.54 [1.10–2.14]). This result remained when combined with eight case-control studies (RR: 1.26 [1.12–1.42]). Furthermore, a positive association was observed for animal fat (RR: 1.31 [1.08–1.58]) when cohorts (*n* = 2) and case-control studies (*n* = 3) were combined (not reported separately). Intake of vegetable fat was not associated with risk of non-Hodgkin lymphoma.

#### Liver cancer

An SR from 2015 [81] identified one cohort study investigating the association between self-reported intake of total n-3 PUFA and ALA and risk of liver cancer (~400 cases in a cohort of ~90,000 participants). No association was observed for either total n-3 PUFA (RR: 0.51 [0.20–1.32]) or ALA (RR: 0.70 [0.29–1.71]). However, when results from this cohort were meta-analyzed together with results from one hospital-based case-control study (185/412 cases/controls), n-3 PUFA was inversely associated (RR: 0.49 [0.19–0.79]) but ALA was not (RR: 0.70 [0.30–1.10]). Self-reported intake of fish was however inversely associated in both cohort and case-control studies (combined RR: 0.65 [0.51–0.79], *n* = 11 studies).

#### Gastric cancer

An SR from 2015 (82) identified only one cohort study (from the United States) investigating the association between self-reported intake of fat and risk of gastric cancer (955 cases in a cohort of ~0.5 million participants). No association was observed for total fat, SFA, MUFA, or PUFA. When results from this cohort were meta-analyzed together with results from ~20 case-control studies (highest vs. lowest intake), both total fat (RR: 1.18 [1.00–1.39]) and SFA (RR: 1.31 [1.09–1.58]) were positively associated whereas PUFA was inversely associated (RR: 0.77 [0.65–0.92]).

#### Bladder cancer

One SR from 2019 (83) identified two publications based on cohorts investigating the associations between fat intake and risk of bladder cancer. No association was observed for total fat, SFA, MUFA, or PUFA and risk of bladder cancer. When results from these cohorts were meta-analyzed together with results from nine case-control studies, intake of total fat was positively associated (RR: 1.28 [1.04–1.58]).

#### Endometrial cancer

A meta-analysis from 2016 (84) based on cohort studies observed no strong association (although indicative) between self-reported intake of total fat and risk of endometrial cancer (highest vs. lowest, RR: 0.91 [0.83–1.01]; *n* = 5 cohorts). However, when four of these five cohorts were analyzed in a dose-response meta-analysis (85), total fat intake was inversely associated (RR: 0.95 [0.91–0.98] per 30 g/day increment). There was no clear association (highest vs. lowest) for SFA (RR: 0.91 [0.80–1.03]; *n* = 3 cohorts), PUFA (RR: 0.94 [0.78–1.12]; *n* = 2 cohorts), or LA (RR: 1.08 [0.95–1.23]; *n* = 2 cohorts) (84). However, MUFA was inversely associated (RR: 0.85 [0.73–0.98]; *n* = 3 cohorts). When these same cohorts were instead combined in a dose-response meta-analysis (86), marginal inverse associations were indicated for both SFA (RR: 0.97 [0.93–1.00] per 10 g/day increment) and MUFA (RR: 0.97 [0.94–1.00] per 10 g/day increment), but not PUFA (RR: 1.00 [0.86–1.16] per 10 g/day increment). Cohorts were conducted in Europe, the United States, Norway, and Canada and follow-up ranged between 7 and 21 years. Taken together, although some associations between self-reported intake of fat and risk of endometrial cancer are indicated from meta-analyses (based on relatively few cohorts), potential effects are likely to be small and results should be interpreted cautiously.

#### Colorectal cancer

Based on the most recent (2021) meta-analysis (87), including a total of 26 prospective studies, the risk of colorectal cancer is not associated with self-reported intake of total fat (RR: 1.01 [0.92–1.10]), SFA (RR: 0.98 [0.90–1.06]), MUFA (RR: 1.04 [0.93–1.15]), PUFA (RR: 1.04 [0.92–1.17]), n-6 PUFA (RR: 1.02 [0.93–1.11]), n-3 PUFA (RR: 1.00 [0.91–1.10]), EPA+DHA (RR: 1.03 [0.92–1.13]), n-6:n-3 ratio (RR: 1.10 [0.97–1.25]), or n-3:n-6 ratio (RR: 1.12 [0.96–1.31]). In stratified analyses, no sex-specific, anatomic-specific, or region-specific associations were observed. Regarding individual fatty acids, no association was observed for AA, ALA, and DPA whereas LA was positively associated (RR: 1.19 [1.07–1.33]) and both EPA (RR: 0.88 [0.82–0.95]) and DHA (RR: 0.89 [0.83–0.97]) were inversely associated. The lack of association observed for total fat, SFA, MUFA, and PUFA in this most recent meta-analysis is, overall, supported by previous and contemporary meta-analyses (68, 72, 88–91). Similarly, a meta-analysis from 2020 (68) based on four prospective studies observed no association between blood levels of n-6 PUFA and risk of colorectal cancer (RR: 0.92 [0.77–1.10]).

#### Breast cancer

*Trans fat.* In a meta-analysis from 2020 (92), based on eight effect sizes from six prospective studies, there was no association (highest vs. lowest) between self-reported intake of total trans-fat and risk of breast cancer (RR: 1.02 [0.95–1.10]). Similarly, when analyzed for dose-response, there was no association per 1 g per day increment (RR: 1.00 [0.99–1.01]). Based on three effect sizes, intake of CLA was not associated with risk of breast cancer (RR: 1.05 [0.95–1.17]). Furthermore, serum levels of total TFAs were not associated with risk of breast cancer (RR: 1.01 [0.89–1.14]), based on five effect sizes. However, when removing two effect sizes from studies in premenopausal women, a positive association between serum levels of total TFAs and risk of breast cancer was observed (RR: 1.37 [1.04–1.81]).

*Total fat, SFA, MUFA, PUFA*. In a meta-analysis from 2016 (93), based on effect sizes from 20 prospective studies, self-reported intake of total fat (highest vs. lowest) was positively associated with risk of breast cancer (RR: 1.10 [1.02–1.19]). However, in subgroup analyses, this was only observed in postmenopausal women, only in studies with shorter duration (<10 years), only in Europe (*n* = 8) and only in studies not adjusting for education, BMI, reproductive variables, and family history of breast cancer. Risk of breast cancer was not associated (although indicative of harm) with self-reported intake (highest vs. lowest) of SFA (RR: 1.08 [0.99–1.18], *n* = 20), MUFA (RR: 1.08 [0.97–1.21], *n* = 17), or PUFA (RR: 1.05 [0.95–1.14], *n* = 16). Similarly, there was no association between self-reported intake of n-3 PUFA (RR: 1.02 [80.89–1.17], *n* = 5), n-6 PUFA (RR: 1.10 [0.88–1.38], *n* = 5), EPA (RR: 0.93 [0.79–1.11], *n* = 4), DHA (RR: 0.94 [0.77–1.16], *n* = 4), ALA (RR: 0.99 [0.92–1.07], *n* = 5), LA (RR: 1.01 [0.94–1.08], *n* = 9), AA (RR: 1.00 [0.93–1.08], *n* = 5), and risk of breast cancer. Similarly, serum levels of PUFA (RR: 0.59 [0.27–1.30], *n* = 4), n-3 PUFA (RR: 0.81 [0.60–1.10], *n* = 6), and n-6 PUFA (RR: 0.84 [0.60–1.18], *n* = 7) were not associated with risk of breast cancer.

In a meta-analysis from 2017 (94) looking specifically at breast cancer mortality, there was no association for consumption of total fat when highest versus lowest categories were compared (HR: 1.14 [0.86–1.52], *n* = 6 cohorts) or when assessed per 20 g/day increments (HR: 1.03 [0.97–1.10], *n* = 4 cohorts). However, intake of SFA was positively associated with breast cancer mortality when highest versus lowest intake categories were compared (HR: 1.51 [1.09–2.09], *n* = 4 cohorts) but not when assessed linearly per 20 g/day increments (HR: 1.03 [0.77–1.38], *n* = 4 cohorts). Results did not change markedly in sensitivity analyses examining the impact of timing of dietary assessment (i.e. pre/post diagnosis).

A meta-analysis from 2013 (95), including 17 articles based on prospective studies, observed an inverse association between self-reported intake (highest vs. lowest) of marine n-3 PUFA and risk of breast cancer (RR: 0.85 [0.76–0.96]). The estimated effect size was similar when based only on studies (*n* = 8) using tissue biomarkers (RR: 0.86 [0.71–1.03]). When studies using self-reported intake data were combined with studies using tissue biomarkers, the relative risk was 0.86 (0.78–0.94). When analyzed for dose response, the relative risk of breast cancer was 0.95 (0.90–1.00), both when expressed per 0.1 g/day increment (*n* = 3) and per 0.1% energy/day increment (*n* = 6). Self-reported intake of ALA (*n* = 12) was not associated with risk of breast cancer, either when compared as ‘highest versus lowest’ (RR: 0.97 [0.90–1.04]) or in dose-response analysis (RR: 0.99 [0.98–1.01] per 0.1 g/day increment; RR: 1.00 [0.99–1.00] per 0.1% energy/day increment). Similarly, self-reported intake of total n-3 PUFA (*n* = 10) was not associated with risk of breast cancer (RR: 0.96 [0.86–1.06]). The latest meta-analysis from 2021 (72) supports these findings, observing an inverse association (highest vs. lowest) for marine n-3 PUFA (RR: 0.70 [0.55–0.91], *n* = 5) but no association for total n-3 PUFA (RR: 1.02 [0.91–1.15], *n* = 7) or ALA (RR: 0.99 [0.92–1.07], *n* = 5).

A meta-analysis from 2020 (68) based on 13 prospective studies observed no association between self-reported intake of n-6 PUFA and risk of breast cancer (RR: 1.00 [1.00–1.01]); however, blood levels of n-6 PUFA were inversely associated (RR: 0.87 [0.77–0.98], *n* = 11 cohorts). The latest meta-analysis from 2021 (72), although based on a combination of prospective and case-control studies, supports the lack of association for self-reported intake of n-6 PUFA (highest vs. lowest, RR: 1.12 [0.97–1.29], *n* = 9).

The dietary intake ratio of n-3:n-6 PUFA was not associated with risk of breast cancer (96).

#### Lung cancer

An SR and dose-response meta-analysis from 2021 (97) based on eight effect sizes from five prospective cohort studies found no association between dietary intake of PUFA and risk of lung cancer (RR: 0.99 [0.96–1.01]), with follow-up duration ranging from 7 to 24.8 years.

#### Evidence from randomized trials

*Long-chain n-3 PUFA.* Based on a meta-analysis of randomized trials (98), increasing intake of long-chain n-3 PUFA (primarily through supplements) has little or no effect on diagnosis of any cancer (RR: 1.02 [0.98–1.07]; *n* = 27 trials, mean dose 1.7 g/day, mean duration 32 months). Similarly, increasing long-chain n-3 PUFA probably has little or no effect on cancer death (RR: 0.97 [0.90–1.06]; *n* = 18 trials). For specific cancers, there is probably little or no effect on breast cancer diagnosis (RR: 1.03 [0.89–1.20]; *n* = 12 trials, mean dose 1.9 g/day, mean duration 48 months) but increasing long-chain n-3 PUFA may potentially increase risk of prostate cancer (RR: 1.10 [0.97–1.24]; *n* = 7 trials, mean dose 1.2 g/day, mean duration 51 months). However, the evidence for this potential harm was graded as ‘low quality’.

*ALA.* Based on only two trials (2 and 5 g/day, 24 and 40 month duration), increased intake of ALA does not appear to have any strong effect on cancer death (RR: 1.05 [0.74–1.49]; *n* = 123 cases) but may potentially increase the risk of prostate cancer diagnosis (RR: 1.30 [0.72–2.32]; *n* = 46 cases) (98).

*n-6 PUFA.* Evidence is unclear and of very low quality; no conclusion can be drawn (98).

*Total PUFA.* Based on eight trials (*n* = 436 cases) with a mean duration of 39 months, increasing total PUFA (mean 9.6 E%, median 3.3 E%) may increase risk of any cancer diagnosis (RR: 1.19 [0.99–1.42], graded as ‘low-quality evidence’) but has no clear effect on cancer deaths (RR: 1.10 [0.48–2.49], *n* = 4 trials and 73 deaths; mean 13 E%, median 7 E%). Doses varied considerably between studies but subgroup analyses did not suggest differences due to PUFA dose (98).

### Concluding remarks

Taken together, there are no strong associations between self-reported intake of total fat or the quality of fat and risk of individual cancers, but the evidence is inconclusive and inconsistent. Results from biomarker studies indicate that higher blood/tissue levels of marine n-3 PUFA may be associated with lower risk of breast cancer as well as lower risk of cancer mortality overall. Higher levels of n-6 PUFA may be associated with a lower risk of any cancer.

#### Osteoporosis and bone health

An SR from 2012 of RCTs (99) investigating the effect of n-3 PUFA supplements/enriched diets on bone mineral density (BMD) and bone turnover markers identified 10 studies. No pooled analysis was performed due to heterogeneous populations and interventions. Of the four studies reporting on BMD, three reported no difference between n-3 PUFA and control whereas one observed that n-3 PUFA combined with calcium was beneficial compared to SFA combined with calcium for both lumbar and femoral neck BMD. For bone turnover markers, two studies reported no difference between n-3 PUFA and control; one study observed that dairy products fortified with ALA, EPA, and DHA may decrease a urinary marker of bone resorption compared to standard dairy products; one study observed that milk fortified with fish oil, oleic acid, and vitamins may improve regulators of bone turnover compared to standard milk; and one study observed that a high ALA diet may decrease a marker of bone resorption compared to average American diet.

A meta-analysis from 2017 (100) of RCTs investigating the effect of n-3 PUFA supplements on bone turnover markers specifically in postmenopausal women identified eight studies. n-3 PUFA decreased serum osteocalcin compared to control (−0.86 [−1.68 to −0.04], *I*^2^ = 0.0%), whereas there were no effects on bone-specific alkaline phosphatase (BALP) (−0.08 [−0.29 to 0.12], *I*^2^ = 0.0%) or collagen type 1 cross-linked C-telopeptide (CTX) (−0.00 [−0.04 to 0.04], *I*^2^ = 0.0%).

Based on a meta-analysis from 2019 (101) of prospective cohort studies with follow-up ranging from 7 to 24 years, higher compared to lower intake of total n-3 PUFA fatty acids was associated with a 12% lower risk of hip fracture (0.88 [0.80–0.98]) without heterogeneity, based on six effect sizes from four cohorts. No association was observed for ALA (1.01 [0.90–1.13] or EPA+DHA (0.91 [0.81–1.03]) specifically.

A qSR and meta-analysis from 2019, based on RCTs, investigated the effects of n-3, n-6, and total PUFA on various measures of bone health (102). The included studies were a mix of studies performed in healthy participants and in specific patient populations. The effect of increasing n-3 PUFA on fracture risk is unclear (RR: 0.16 [0.01–3.92], based on a single RCT including 126 participants for 26 weeks duration; very low evidence grade). Increasing n-3 PUFA may slightly increase lumbar BMD (0.03 g/cm^2^ [−0.02 to 0.07], based on four RCTs; low evidence grade) but effects on femoral neck BMD (0.04 g/cm^2^ [0–0.08], based on four RCTs; very low evidence grade) and total proximal femur BMD (0.03 g/cm^2^ [−0.3 to 0.36], based on one RCT; very low evidence grade) are unclear. Based on a single RCT with 37 participants for 11 months duration, increasing n-3 PUFA may have little or no effect on total bone mass (0.2 kg [−2.8 to 3.2], low evidence grade). Similarly, increasing total PUFA may have little or no effect on BMD (based on three RCTs, low evidence grade).

In a meta-analysis of observational studies from 2018 (103), intake of total fat (higher compared to lower) was not associated with risk of total fractures either in prospective studies (*n* = 2) or case-control studies (*n* = 4). Intake of SFA was positively associated with hip fracture in one prospective study (1.31 [1.11–1.55]) as well as when combined with two case-control studies (1.79 [1.05–3.03]). Intake of total MUFA was not associated with risk of total fractures (1.47 [0.74–2.92]) but MUFA derived from animal sources was positively associated in subgroup analysis (2.29 [1.50–3.50]).

### Concluding remarks

Data on the potential link between dietary fat and osteoporosis are limited and no strong conclusion can be drawn. The available evidence is focused on n-3 PUFA and indicates overall protective effects.

#### Mental health

In a qSR from 2020 (104) including RCTs only (38 RCTs, 41 comparisons, 49,757 participants), meta-analysis suggested no or very little effect of long-chain n-3 PUFA on new neurocognitive illness (RR: 0.98 [0.87–1.10], six RCTs, 33,496 participants, *I*^2^ = 36%), new cognitive impairment (RR: 0.99 [0.92–1.06], five RCTs, 33,296 participants, *I*^2^ = 0%), or global cognition assessed using the Mini-Mental State Examination (MD: 0.10 [0.03–0.16], 13 RCTs, 14,851 participants, *I*^2^ = 0%), all moderate-quality evidence. Effects did not differ with sensitivity analyses, and no differential effects by dose, duration, intervention type, or replacement were found. Effects of increasing ALA, n-6, or total PUFA were unclear. The authors conclude that the data set enabled assessment of effects on neurocognitive illness and cognitive decline and they found that long-chain n-3 PUFA probably has little or no effect on new neurocognitive outcomes or cognitive impairment so that long-chain n-3 PUFA supplements do not help older adults protect against cognitive decline.

The *de novo* NNR2023 SR addressed the relation between dietary fat quality and risk of Alzheimer disease (AD) and dementia in adults aged 50 years and older (105). Five articles, based on four prospective cohorts (*n* = 2 conducted in the United States, *n* = 1 in Finland, and *n* = 1 in the Netherlands) with a minimum of 5 years of follow-up were included. A total of 530,576 participants, mainly healthy from the general population, were included in the analysis and the mean age range at baseline was 63–76 years. Semi-quantitative FFQs were used in all studies to assess dietary intake. Three of the studies had serious risk of bias while one had moderate risk. Findings were partly contradictory, but overall, no robust associations between dietary fat quality and development of AD or dementia were found. The evidence for SFA, MUFA, PUFA, and TFA were all considered as ‘limited-no conclusion’. Similarly, results for subclasses such as plant- or animal-derived MUFA and n-6 or n-3 PUFA were generally nonsignificant.

In a qSR and meta-analysis from 2021, including RCTs only, Deane et al. assessed the effects of increasing PUFA in adults for at least 24 weeks on prevention of depression (106). In the main analysis (*n* = 13 studies), including both healthy populations and populations with preexisting disease, there was no effect (RR: 1.01 [0.92–1.10]) but subgroup analysis based only on healthy populations (*n* = 3 studies including *n* = 1250 participants) suggested increased depression risk when increasing long-chain n-3 PUFA (RR: 1.35 [1.02–1.79]).

### Concluding remarks

Data on the potential link between dietary fat quality and risk of AD and dementia are limited and no strong conclusion can be drawn. The available evidence does not suggest any robust associations for any of the fatty acid classes.

#### Other health outcomes and risk markers

*Inflammatory bowel disease.* In a qSR from 2021, Ajabnoor et al. (107) studied long‑term effects of increasing n‑3, n‑6, and total PUFA on inflammatory bowel disease (IBD) and markers of inflammation. Only RCTs were included, in total 83 RCTs (41,751 participants), of which 13 recruited participants with IBD. The authors found that increasing n-3 PUFA may reduce risk of IBD relapse (RR: 0.85 [0.72–1.01]) and IBD worsening (RR: 0.85 [0.71–1.03]) and reduce erythrocyte sedimentation rate (ESR, SMD: −0.23 [−0.44 to −0.01]), but may increase IBD diagnosis risk (RR: 1.10 [0.63–1.92]) and fecal calprotectin, a specific inflammatory marker for IBD (MD: 16.1 μg/g [−37.6 to 69.8], all low-quality evidence). Outcomes for ALA acid, n-6, and total PUFA were sparse, but suggested little or no effect where data were available. The authors concluded that supplementation with PUFAs has little or no effect on prevention or treatment of IBD and provides little support for modification of long-term inflammatory status.

*Liver fat content and nonalcoholic fatty liver disease.* An SR and meta-analysis from 2021 reviewed the effects of dietary macronutrients on liver fat content in adults (108). The authors found that replacing dietary fat with carbohydrates did not result in changes in liver fat (SMD: 0.01 [−0.36 to 0.37], **n** = 12 comparisons). However, unsaturated fat (primarily n-6 PUFA) reduced liver fat compared with saturated fat (−0.80 [−1.09 to −0.51], **n** = 4 comparisons). The protective effect of dietary n-6 PUFA (i.e. LA) has been shown in both isocaloric (109) and hypercaloric (110, 111) settings and also been indicated in prospective observational analyses using biomarkers where higher serum levels of LA were associated with markedly reduced risk of developing NAFLD during follow-up (112, 113).

*Semen quality.* In an SR from 2019 (114), 16 RCTs and observational studies on infertile and fertile men were included evaluating the effect of n-3 fatty acids or dietary fish intake on semen quality. The studies were very heterogeneous in participants (fertile/infertile, age, BMI, ethnicity, etc.), and no meta-analysis was performed. The authors conclude that n-3 PUFA supplements and dietary intake of n-3 PUFA might improve semen quality parameters in infertile men and men from couples seeking fertility treatment.

*Inflammation, oxidative stress, and vascular health.* Pase et al. (115) reviewed if long-chain n-3 fatty acids reduce arterial stiffness. By using pulse wave velocity or arterial compliance, n-3 fatty acids were statistically significant in effectively improving both pulse wave velocity and arterial compliance.

Wang et al. (116) reviewed the effect of n-3 fatty acid supplementation on endothelial function. They concluded that supplementation of n-3 fatty acids significantly improves the endothelial function without affecting endothelium-independent dilation, as measured by flow-mediated dilation and endothelium-independent vasodilation.

Li et al. (117) reviewed the effect of marine-derived n-3 PUFA on C-reactive protein (CRP), interleukin 6 (IL-6), and tumor necrosis factor α (TNF-α). They concluded that marine-derived n-3 PUFA supplementation had a significant lowering effect on CRP, IL-6, and TNF-α level.

An SR and meta-analysis from 2019 of RCTs investigated the effects of n-3 PUFA supplementation compared with placebo on oxidative stress parameters, although the majority of studies were performed in various patient groups (118). n-3 PUFA supplementation reduced malondialdehyde (SMD: −0.42 [−0.62 to −0.21], *I*^2^ = 74%, *n* = 25 trials) and increased serum total antioxidant capacity (SMD: 0.48 [0.23 to 0.72], *I*^2^ = 60%, *n* = 11 trials) and glutathione peroxidase (SMD: 0.73 [0.30 to 1.16], *I*^2^ = 83%, *n* = 10 trials). n-3 PUFA supplementation had no effect on serum levels of nitric oxide (SMD: −0.17 [−0.77 to 0.43], *I*^2^ = 91%, *n* = 10 trials), reduced glutathione (SMD: 0.23 [−0.17 to 0.64], *I*^2^ = 75%, *n* = 7 trials), superoxide dismutase (SMD: 0.12 [−0.40 to 0.65], *I*^2^ = 89%, *n* = 10 trials), or catalase (SMD: 0.16 [−0.33 to 0.65], *I*^2^ = 75%, *n* = 6 trials).

No qSR on the effects of n-6 PUFA on inflammation was available and no SR was identified through the search. However, one SR from 2011, based on 11 comparisons from human intervention trials, found that decreases (−12 to −90%) in dietary intake of LA were not associated with changes in AA in plasma phospholipids (119). Similarly, increases (12–550%) in dietary LA were not associated with changes in AA in plasma phospholipids. Furthermore, one SR from 2012, based on 15 human intervention trials, found no effect of dietary LA on a wide variety of inflammatory markers (120). Thus, based on available evidence from trials in humans, increasing dietary LA (the major dietary PUFA) does not appear to exert detrimental effects on inflammation.

*Postprandial metabolism.* Kdekian et al. (121) reviewed the impact of isocaloric exchanges of carbohydrate for fat on postprandial glucose, insulin, triglycerides, and free fatty acid responses. The found that for each 10 E% increase in fat, replacing carbohydrates produced a mean reduction in postprandial glucose of 0.32 mmol/L (−0.64 to −0.00), a reduction in insulin of 18.2 pmol/L (−24.86 to −11.54), and an increase in triglycerides of 0.06 mmol/L (0.02 to 0.09), with no statistically significant effect on free fatty acids.

An SR from 2020 (122) evaluated the effects of dietary fat composition on metabolic endotoxemia. Of the 11 RCTs identified, eight were acute meal tests and reported only on postprandial effects, whereas the remaining three studies had a duration between 4 and 12 weeks. In acute settings, intake of SFA increased whereas intake of PUFA decreased postprandial lipopolysaccharide (LPS), but no effects of dietary fat composition on LPS were observed in the longer-term studies.

Muscle mass and function. In a qSR and meta-analysis from 2019, based on RCTs with at least 24 weeks duration, Abdelhamid et al. (102) investigated the effects of n-3, n-6, and total PUFA on various measures of muscle mass and functional status. The included studies were a mix of studies performed in healthy participants and in specific patient populations. Based on seven RCTs including 476 participants, the effect of increasing n-3 PUFA on muscle mass (various measures combined) is unclear as the evidence is of very low quality. Similarly, increasing n-3 PUFA may have little or no effect on functional status (various measures combined), with low evidence grade. Based on two RCTs, increasing n-3 PUFA did not suggest a significant effect on handgrip strength; however, it did suggest a positive effect on physical performance (measured as leg extensor power, walking speed, and repeated chair rises) based on 161 participants. Increasing total PUFA may have little or no effect on muscle mass/fat-free mass, based on a single RCT including 214 participants, with low evidence grade.

An SR and meta-analysis from 2020 (123) of RCTs (with duration 10–24 weeks) investigating the effects of n-3 PUFA on muscle mass, strength, and performance in individuals >60 years of age identified 10 studies. The included studies were primarily performed in healthy participants. Increasing n-3 PUFA improved ‘time up and go test’ (−0.30 [−0.43 to −0.17], *I*^2^ = 37%) based on four RCTs including 136 participants, with very low evidence grade. Increasing n-3 PUFA also increased muscle mass (0.33 [0.05–0.62], *I*^2^ = 0%) based on six RCTs including 202 participants, with moderate evidence grade. However, increasing n-3 PUFA had no effect on grip strength (0.53 [−0.64 to 1.69], three RCTs including 97 participants), one-repetition maximum leg strength (−0.15 [−0.93 to 0.62], three RCTs including 88 participants), or walking speed (0.81 [−0.05 to 1.67], five RCTs including 251 participants), all with very low evidence grade. A scoping SR from 2021 (124) observed a positive effect of n-3 PUFA supplementation on lean body mass in healthy populations (0.28 [0.07 to 0.48], *n* = 12 studies) but not in disease populations (primarily cancer patients). There was no effect on handgrip strength (*n* = 7 studies in healthy) but a positive effect was observed on maximal voluntary capacity (*n* = 8 studies in healthy). Finally, an SR and meta-analysis from 2022 of observational studies (125) observed an inverse association between dietary n-3 PUFA (highest vs. lowest) and sarcopenia (OR: 0.41 [0.26 to 0.65], *n* = 6 studies, *I*^2^ = 60%) in adjusted models. No association was observed for dietary n-6 PUFA (highest vs. lowest; OR: 0.64 [0.33 to 1.24], *I*^2^ = 66.8%).

Chronic obstructive pulmonary disease. An SR from 2014 (126), based on three cross-sectional and two case-control studies, investigated the association between types of dietary fat and chronic obstructive pulmonary disease. The limited evidence was inconsistent, precluding any firm conclusions.

#### Total mortality

In the qSR by Hooper from 2020 investigating the effect of reduction in SFA intake for CVD prevention (49), no effect on all-cause mortality (RR: 0.96 [0.90–1.03], *I*^2^ = 2%, based on 12 trials) was found for diets lower compared to higher in SFA. This lack of effect remained in subgroup analyses where SFA was replaced by PUFA (RR: 0.96 [0.82–1.13], *I*^2^ = 26%, *n* = 7 trials), MUFA (RR: 3.00 [0.33–26.99], *n* = 1 trial), carbohydrates (RR: 0.97 [0.90–1.04], *I*^2^ = 0%, *n* = 6 trials), and protein (RR: 0.97 [0.90–1.04], *I*^2^ = 0%, *n* = 5 trials).

Using observational studies, de Souza et al. (50) reported that SFA intake was not associated with all-cause mortality (RR: 0.99 [0.91 to 1.09], *I*^2^ = 33%, *n* = 6 prospective studies). Higher intake of total TFA was positively associated with all-cause mortality (RR: 1.34 [1.16 to 1.56], *I*^2^ = 70%, *n* = 2 studies), but neither industrial TFAs (RR: 0.98 [0.92 to 1.04], *I*^2^ = 0%) nor ruminant TFAs (RR: 1.04 [0.92 to 1.18], *I*^2^ = 4%) were associated with all-cause mortality.

Reynolds et al. (54) performed a SR and meta-analysis of 112 publications (3,696, 568 participants) relating to SFA. They found that mortality was reduced when 5 E% of SFA was replaced with PUFA (RR: 0.85 [0.75 to 0.97]), MUFA (RR: 0.84 [0.75 to 0.95]), plant MUFA (RR: 0.85 [0.82 to 0.88]), and carbohydrates (RR: 0.92 [0.86 to 0.99]). Tissue levels of 15:0 (RR: 0.99 [0.90 to 1.09]) and 17:0 (RR: 0.97 [0.81 to 1.16]) were not associated with total mortality. Thirteen publications (providing information on 184,397 deaths in 770,780 people) were available for TFA. Higher (compared to lower) intake of total TFA was associated with increased risk of total mortality (RR: 1.11 [1.02 to 1.20]) and intake >1% of total energy was associated with total mortality when compared to intake <1% of total energy (RR: 1.11 [1.00 to 1.24]). Tissue levels of TFA were not associated with total mortality, or total TFA, individual isomers, ruminant TFA, or industrial TFA.

*Results from non-qualified systematic reviews and biomarker studies*. An SR and meta-analysis from 2021 (40), based on 19 prospective cohort studies including 1,013,273 participants and 195,515 deaths, found an inverse associations between all-cause mortality and a 5 E% increment in intakes of total fat (RR: 0.99 [0.98–1.00]), MUFA (RR: 0.98 [0.97–0.99]), and PUFA (RR: 0.93 [0.89–0.97]). A 1 E% increment in dietary TFA was associated with 6% higher risk of mortality from all-causes (RR: 1.06 [1.01–1.10]), and SFA was nonlinearly positively associated with all-cause mortality up to 11 E%, reaching a plateau at higher intakes.

In an SR and meta-analysis from 2021 of prospective cohort studies (43), intake of MUFA (highest vs. lowest) was inversely associated with all-cause mortality (RR: 0.94 [0.90–0.98], *I*^2^ = 52.4%, *n* = 15 effect sizes from 11 studies including a total of 191,283 deaths). Dose-response analyses indicated that each 5 E% MUFA was associated with a 3% decreased risk of all-cause mortality (RR: 0.97 [0.96–0.98]).

An SR and meta-analysis from 2021 (52) of prospective studies investigated the association between circulating or adipose tissue levels of 15:0, 17:0, and t16:1n-7 (potentially partly reflecting dairy fat intake) and all-cause mortality. Limited evidence was available, and none of the fatty acids (highest vs. lowest) were associated with all-cause mortality (RR: 0.98 [0.81–1.20] for 15:0, *n* = 3 studies including a total of 3,709 deaths; RR: 0.91 [0.70–1.19] for 17:0, *n* = 2 studies including a total of 3,003 deaths; and RR: 1.07 [0.97–1.17] for t16:1n-7, *n* = 1 study including 2,428 deaths).

An individual-level pooled analysis from 2021 studied circulating levels of n-3 PUFA and risk of all-cause mortality (38). Among 17 prospective cohorts with a median of 16 years of follow-up, higher compared to lower levels of EPA (HR: 0.82 [0.78–0.87]), DPA (HR: 0.84 [0.79–0.90]), and DHA (HR: 0.85 [0.81–0.90]) were associated with lower risk for all-cause mortality.

### Concluding remarks

Based on both trials and observational studies, reducing intake of SFA does not influence the risk of all-cause mortality. Data from non-qSRs and meta-analyses indicate that both MUFA and PUFA are marginally inversely associated with all-cause mortality. Biomarker studies indicate that higher levels of marine n-3 PUFA are inversely associated with all-cause mortality.

#### Mother and child health

*Neurodevelopment, allergies, birth weight, and length of gestation.* In a qSR from 2016, Newberry et al. (127) included 95 RCTs using n-3 PUFA in pregnant or breastfeeding women or neonates. They also included 48 prospective observational studies that analyzed the association between baseline n-3 PUFA intake or biomarker level and follow-up outcomes. Most studies examined the effects of fish oil (or other combinations of DHA and EPA) supplements on pregnant or breastfeeding women, or the effects of infant formula fortified with DHA plus AA. The authors conclude that, with the exception of small increases in birth weight and length of gestation, n-3 PUFA supplementation or fortification has no effects on peripartum maternal or infant health outcomes. No effects of n-3 PUFA were seen on gestational hypertension, peripartum depression, or postnatal growth. Apparent effects of n-3 PUFA supplementation were inconsistent across assessment methods and follow-up times for outcomes related to infant visual acuity, cognitive development, and prevention of allergy and asthma.

In an SR from 2015, Delgado-Noguera et al. (128) assessed the effectiveness and safety of supplementation with long-chain PUFA in breastfeeding mothers in the cognitive and physical development of their infants as well as safety for the mother and infant. They included eight RCTs involving 1,567 women. The longest follow-up was 7 years. No significant difference in children’s neurodevelopment at long-term follow-up beyond 24 months was found: language development (SMD: −0.27 (−0.56 to 0.02); two trials, 187 participants); intelligence or problem-solving ability (three trials, 238 participants; SMD: 0.00 (−0.36 to 0.36); psychomotor development (SMD: −0.11 [−0.48 to 0.26]; one trial, 113 participants); motor development (SMD −0.23 [−0.60 to 0.14]; one trial, 115 participants); or in general movements (RR: 1.12 [0.58 to 2.14]; one trial, 77 participants; at 12 weeks of life). For child visual acuity, there was no significant difference (SMD: 0.33 [−0.04 to 0.71]; one trial, 111 participants). No adverse effects were reported. The authors concluded that supplementation did not appear to improve children’s neurodevelopment, visual acuity, or growth. In child attention at 5 years of age, weak evidence was found (one study) favoring supplementation.

The DGAC 2020 (129) investigated the relationship between n-3 PUFA supplementation during pregnancy and lactation and developmental milestones, including neurocognitive development, in the child. A total of 31 articles (from 14 RCTs and one prospective cohort) were included; the majority of articles (*n* = 25) investigated supplementation during pregnancy only. For cognitive development, five of eight studies found a favorable effect of supplementation on at least one measure. Based on this, the authors conclude that there is limited evidence to suggest that n-3 PUFA supplementation during pregnancy may result in favorable cognitive development in the child. Furthermore, the authors conclude that there is insufficient evidence to determine the relationship between n-3 PUFA supplementation and language and social emotional development, motor and visual development, academic performance and risk of attention-deficit disorder (ADD), attention-deficit hyperactivity disorder (ADHD), and autism spectrum disorder. No evidence was available to determine the relationship between n-3 PUFA supplementation and anxiety or depression.

An SR from 2020 investigated the effect of infant formula with or without long-chain PUFA on long-term cognitive function in childhood (130). The authors included eight trials, with age at the last available cognitive test ranged from 3.3 to 16 years. The overall quality of the evidence was low, and no significant effects were observed. The authors concluded that the effect of long-chain PUFA supplementation in infants on cognition is highly uncertain and includes potential for large benefit as well as large harm and cannot thus be recommended until further evidence excludes long-term harm.

Collectively, based on the three qSRs described earlier, there is inconclusive evidence to support or refute the practice of giving n-3 PUFA supplementation to pregnant and/or breastfeeding mothers in order to improve neurodevelopment or visual acuity.

An SR and meta-analysis from 2016 of RCTs investigated if n-3 PUFA supplements could prevent early (<34 weeks) and any (<37 weeks) preterm delivery (131). Nine RCTs were included (a total of 5,980 women). In the majority of studies, the intervention commenced before 24 weeks of gestation and doses ranged from 133 to 3,000 mg/day. n-3 PUFA supplements reduced the risk of both early (RR: 0.42 [0.27–0.66], *I*^2^ = 0%, *n* = 6 studies) and any preterm (RR: 0.83 [0.70–0.98], *I*^2^ = 0%, *n* = 9 studies) delivery.

An SR and meta-analysis from 2018 of RCTs investigated if maternal n-3 PUFA supplementation influenced body composition in the offspring (132). Twenty-six RCTs were included (a total of 10,970 participants); dose of DHA ranged between 200 and 1,183 mg per day and dose of EPA ranged between 0 and 1,280 mg per day. Length of follow-up ranged between 0 and 19 years. Maternal n-3 PUFA supplementation had no effects on offspring BMI (0.09 kg/m^2^ [−0.05 to 0.23], *I*^2^ = 42.9%, *n* = 8 studies), sum of skinfold thickness (0.45 mm [−0.30 to 1.20], *I*^2^ = 0%, *n* = 4 studies), fat mass (0.05 kg [−0.01 to 0.11], *I*^2^ = 36.2%, *n* = 3 studies), or percentage of body fat (0.04 [−0.38 to 0.46], *I*^2^ = 22.9%, *n* = 5 studies) but increased waist circumference (0.35 cm [0.04 to 0.67], *I*^2^ = 37.1%, *n* = 4 studies).

An SR and meta-analysis from 2021 investigated the effects and associations of n-3 PUFA and TFA during pregnancy and offspring body weight during childhood (133). Nineteen RCTs (27 studies) and 14 observational studies were included. In a meta-analysis of RCTs, maternal n-3 PUFA supplementation had no effect on offspring body weight (−0.05 [−0.3 to 0.2]) or BMI (0.08 [−0.3 to 0.4]) at 0–4 years of age, irrespective of dose. At offspring age 5–10 years, maternal n-3 PUFA supplementation had no effect on body weight but slightly increased BMI compared to the control group. In observational studies, levels of TFA in maternal blood, cord blood, or placental tissue were related to lower birth weight in three studies, while one study found no association.

An SR and meta-analysis from 2020 of RCTs investigated the effects of n-3 PUFA supplementation on metabolic status in pregnant women (134). Fourteen studies were included (a total of 1,468 participants) with duration ranging from 6 to 25 weeks. n-3 PUFA supplementation increased HDL-C (3.10 [0.18–6.03], *I*^2^ = 87.2%, *n* = 6 studies) and decreased CRP (−1.85 [−2.61 to −1.09], *I*^2^ = 28%, *n* = 4 studies) but had no effect on fasting glucose (0.11 [−2.52 to 2.74], *I*^2^ = 71.8%, *n* = 6 studies), insulin (−0.79 [−2.24 to 0.66], *I*^2^ = 54.3%, *n* = 6 studies), HOMA-IR (−0.56 [−1.38 to 0.26], *I*^2^ = 80.3%, *n* = 4 studies), total cholesterol (5.36 [−2.83 to 13.56], *I*^2^ = 77.3%, *n* = 7 studies), triglycerides (−8.38 [−27.01 to 10.24], *I*^2^ = 88.4%, *n* = 6 studies), LDL-C (11.98 [−0.04 to 24.00], *I*^2^ = 78.1%, *n* = 5 studies), total cholesterol/HDL-C ratio (−0.15 [−0.35 to 0.06], *I*^2^ = 73%, *n* = 3 studies), IL-6 (−5.40 [−13.87 to 3.08], *I*^2^ = 98.7%, *n* = 4 studies), IL-8 (0.09 [−0.54 to 0.71], *I*^2^ = 0%, *n* = 3 studies), or malondialdehyde (−0.39 [−1.43 to 0.66], *I*^2^ = 90.1%, *n* = 3 studies).

In an SR from 2015, Gunaratne et al. (135) studied if maternal prenatal and/or postnatal n-3 long-chain PUFA supplementation can prevent allergies in early childhood. More specifically, the objective was to assess the effect of n-3 PUFA supplementation in pregnant and/or breastfeeding women on allergy outcomes (food allergy, atopic dermatitis [eczema], allergic rhinitis [hay fever], and asthma/wheeze) in their children. They included RCTs evaluating the effect of n-3 PUFA supplementation of pregnant and/or lactating women (compared with placebo or no treatment) on allergy outcomes of the infants or children. Trials using a crossover design and trials examining biochemical outcomes only were not included. The authors included eight trials involving 3,366 women and their 3,175 children were included in the review. In these trials, women were supplemented with n-3 PUFA during pregnancy (five trials), lactation (two trials), or both pregnancy and lactation (one trial). The daily dose of n-3 PUFA varied between 400 and 4,500 mg. All trials randomly allocated women to either a n-3 PUFA supplement or a control group. The authors found that n-3 PUFA supplementation showed a clear reduction in the primary outcome of any allergy (medically diagnosed IgE mediated) in children aged 12–36 months (RR: 0.66 [0.44 to 0.98]; two RCTs; 823 children), but not beyond 36 months (RR: 0.86 [0.61 to 1.20]; one RCT, 706 children). For any allergy (medically diagnosed IgE mediated and/or parental report), no clear differences were seen in children either at 12–36 months (RR: 0.89 [0.71 to 1.11]; two RCTs, 823 children) or beyond 36 months of age (RR: 0.96 [0.84 to 1.09]; three RCTs, 1,765 children). The overall conclusion was that the results showed little effect of maternal marine n-3 PUFA supplementation during pregnancy and/or breastfeeding for the reduction of allergic disease in the children. However, there were reductions in some outcomes such as food allergy during the baby’s first year and eczema with marine n-3 PUFA supplementation in women with a baby at high risk of allergy. Currently, there is not enough evidence to say that n-3 PUFA supplements from marine origin during pregnancy and/or breastfeeding for mothers will reduce allergies in their children.

An SR and meta-analysis from 2015 (136) of RCTs investigated whether n-3 PUFA supplementation in children could prevent asthma. Five RCTs were included (a total of 2,415 children). The average age of administration was 0–12 months and average duration of follow-up was 3.5 years (ranging from 6 months to 8 years). n-3 PUFA supplementation had no effect on incidence of asthma (OR: 0.97 [0.65–1.47], *I*^2^ = 52.2%).

Similar to the two SRs described earlier, the NNR2023 *de novo* SR investigated whether supplementation with long-chain n-3 fatty acids during pregnancy, lactation, or infancy reduces the risk of asthma and atopic disease during childhood (137). A total of 18 articles, based on nine RCTs, were included, of which six were conducted during pregnancy, two during infancy, and one during both pregnancy and infancy. Five RCTs included only infants or women with high hereditary risk of developing atopic disease. The included RCTs were performed in Sweden, Denmark, Australia, Mexico, and the United States. For RCTs conducted during pregnancy, interventions started in mid-pregnancy and continued throughout pregnancy. Doses of long-chain n-3 fatty acids ranged from 0.4 to 3.7 g per day, DHA from 0.4 to 2.1 g per day, and EPA from 0 to 1.5 g per day. Meta-analyses showed that supplementation during pregnancy reduced the risk of asthma/wheeze (RR: 0.62 [0.34–0.91], *I*^2^ = 67.4%, *n* = 7 studies, strength of evidence ‘*limited-suggestive’*), but not eczema/atopic dermatitis (RR: 0.86 [0.50–1.22], *I*^2^ = 56.9%, *n* = 5 studies, strength of evidence ‘*limited-inconclusive’*), food allergy (RR: 0.63 [0.06–1.20], *I*^2^ = 29.2%, *n* = 4 studies, strength of evidence ‘*limited-inconclusive’*), or allergic sensitization or atopy (RR: 0.82 [0.51–1.14], *I*^2^ = 53.6%, *n* = 4 studies, strength of evidence ‘*limited-inconclusive’*). Supplementation during lactation or infancy showed no effects on any outcome (strength of evidence ‘*limited-no conclusion’*).

An SR from 2018 investigated the association between PUFA in breast milk and/or colostrum and allergic disease outcomes in children (138). A total of 18 papers were included (14 based on birth cohorts, two case-control, and two cross-sectional). Sample sizes in cohort studies ranged from 34 to 352. For eczema, levels of n-3 PUFA were protective in three studies whereas there was no association in seven studies. Levels of n-6 PUFA were harmful in one study and not associated in seven studies. The n-6:n-3 PUFA ratio was associated with increased risk for eczema in one study and not associated in five studies. For sensitization, levels of n-3 PUFA showed protective associations in four studies, harmful association in one study, and no association in five studies. Levels of n-6 PUFA showed protective associations in two studies and no association in seven studies and the n-6:n-3 PUFA ratio was not associated in five studies but positively associated with food sensitization in one study. For asthma/wheeze, levels of n-3 PUFA showed no association in four studies but protective associations in two studies. Levels of n-6 PUFA showed no association in four studies but harmful association in two studies. Overall, the evidence is heterogeneous and insufficient to determine whether PUFA in breast milk and/or colostrum influences the risk of allergic disease in children.

An SR and meta-analysis from 2019 of prospective observational studies investigated the associations between PUFA and ruminant TFA during the first 1,000 days with development of allergic disease (139). Twenty-six studies were included (a total of 18,416 participants) with a median sample size of 708 (ranging from 65 to 4,976). Fatty acid exposure was assessed mainly from maternal dietary reports, blood sample, or breast milk. Exposure to n-3 PUFA was not associated with eczema (OR: 0.92 [0.78–1.08], *n* = 10 studies, *I*^2^ = 52.8%), asthma (OR: 0.89 [0.75–1.05], *I*^2^ = 0%), wheeze (OR: 1.06 [0.63–1.80], *I*^2^ = 53.6%), allergic rhinitis (OR: 1.29 [0.78–2.14], *I*^2^ = 61.9%), or sensitization (OR: 1.04 [0.86–1.27], *I*^2^ = 9.1%), with *n* = 2–0 studies for each. Associations were similar when n-3 PUFA was separated into ALA, EPA, and DHA. Exposure to n-6 PUFA was not associated with eczema (OR: 1.00 [0.90–1.11], *I*^2^ = 49.7%), asthma (OR: 0.97 [0.70–1.35], *I*^2^ = 68.8), wheeze (OR: 0.99 [0.87–1.11], *I*^2^ = 30.2%), allergic rhinitis (OR: 1.03 [0.96–1.10], *I*^2^ = 0%), or sensitization (OR: 1.08 [0.94–1.23], *I*^2^ = 9.4%), with *n* = 2–10 studies for each. Associations for n-6 PUFA were similar when separated into LA and AA, except for a positive association between LA and eczema (OR: 1.08 [1.01–1.15], I^2^ = 0%). For ruminant TFA, exposure to vaccenic acid was inversely associated with eczema (OR: 0.42 [0.25–0.72], *I*^2^ = 0%, *n* = 2 studies), but CLA was not associated (OR: 0.67 [0.34–1.32], *I*^2^ = 44.2%, *n* = 2 studies).

An SR and meta-analysis from 2021 of RCTs investigated the effect of milk fat globule membrane (MFGM) supplementation in children (140). Seventeen publications (10 RCTs) were included (a total of 3,575 participants). Most studies recruited participants below 2 months of age and the duration of the intervention ranged from 3 to 18 months. There were no differences between MFGM-supplemented formula and standard formula for anthropometric outcomes (weight, length, head circumference). For psychomotor development, MFGM-supplemented formula increased scores for hand and eye coordination, performance, and general IQ assessed using the Griffith Mental Development Scale at 6 month compared to standard formula in one study but no differences were observed for the Griffith Locomotor, Personal-Social, Hearing and Speech scores. In one trial where psychomotor development was assessed using the Bayley-III scale, the MFGM-supplemented group showed higher scores in the cognitive domain (but not motor domain) at 12 months compared with the standard formula group. In another trial, higher scores in the cognitive, language, and motor domains were observed at 12 months in the MFGM-supplemented group compared to standard formula, but differences did not persist at 18 months. In a study where children were tested at the age of 4 years, use of language and spontaneous oral expression were improved in children who had consumed MFGM-supplemented formula during the first 18 months of life, compared to standard formula.

*Body weight and composition in children.* In a qSR from 2018, Naude et al. (17) reviewed RCTs in children aged 24 months to 18 years and prospective cohort studies if they related baseline total fat intake to weight or body fatness at least 12 months later. The authors were unable to reach firm conclusions. Limited evidence from three trials that randomized children to dietary counseling or education to lower total fat intake (30 E% or less) versus usual or modified fat intake, but with no intention to reduce weight, showed small reductions in BMI, total-, and LDL-C at some time points with lower fat intake compared to controls. There were no consistent effects on weight, HDL-C, or height. Associations in cohort studies that related total fat intake to later measures of body fatness in children were inconsistent and the quality of this evidence was mostly very low.

### Concluding remarks

Based on randomized trials, there is inconclusive evidence to support or refute the practice of giving n-3 PUFA supplementation to pregnant and/or breastfeeding mothers in order to improve neurodevelopment or visual acuity or reduce the risk of allergies in their children. However, supplementation during pregnancy may reduce the risk of asthma and/or wheeze in the offspring, but the strength of evidence is low.

## Requirement and recommended intakes

Recommendations for fat and fatty acids are set in the NNR2023 report (1). A summary of the recommendations is given below.

### Adults

Intake of SFA should be less than 10 E% in the general population (1). Rich sources of SFA include, for example, fatty meat, lard, butter, palm oil, and coconut oil. Detailed recommendations on intakes of specific types of cholesterol-raising SFAs, and cholesterol itself, are not given.

The intake of TFA should be as low as possible (1). Importantly, this applies for both natural TFA in dairy products and industrially produced, partially hydrogenated fats. Typically, a reduction in SFA intake also leads to reduced intake of both TFA and dietary cholesterol.

The intake of MUFA should contribute between 10 and 20 E% in the diet (1).

The intake of n-6 and n-3 PUFA in total should contribute 5–10 E% (1). n-3 PUFA should account for at least 1 E% of the diet. The upper intake range for total PUFA intake is 10 E%. Increased intakes are not recommended because of potential adverse effects.

Intake of MUFA and PUFA should make up at least two-thirds of the total fatty acids (1). Rich sources of unsaturated fat include, for example, nuts and seeds, rapeseed oil, olive oil, and sunflower oil.

Because minimum requirements of PUFA for adults are not known, the estimates are based on threshold intake data from children. The recommendation for essential fatty acids, that is, LA and ALA, is 3 E%, of which at least 0.5 E% should be ALA (1).

For pregnant and lactating women, the contribution of PUFA should be at least 5 E%, including 1 E% from n-3 fatty acids of which at least 200 mg/d should be DHA (1). Pregnant and breastfeeding mothers who eat a vegan or vegetarian diet should be advised to take a daily DHA supplement. When the breastfed child of a vegan mother begins with solid foods, they should also be given DHA supplement.

The evidence for defining an optimal ratio between n-6 and n-3 PUFA is insufficient and no recommendation for the ratio of n-6 to n-3 can be set. If recommendation for intake of n-3 PUFA is met, the ratio is likely of minor importance.

The total fat recommendation is 25–40 E%, which is mainly based on the recommended ranges for fatty acid categories (1). Very low intake of total fat intake (below 20 E%) may lead to difficulties in ensuring sufficient intake of fat-soluble vitamins and essential fatty acids.

By limiting the intake of total fat, a beneficial increase in intake of micronutrients and dietary fiber is typically seen. Furthermore, reduced intake of dietary fat is associated with reduction in body weight, and a reduction in SFA intake in a population results in lower total and LDL-C.

A recommended target of total fat for dietary planning purposes is the middle value of the range, that is, about 32–33 E%.

### Children 6–23 months

Because exclusive breastfeeding is recommended during the first 4–6 months of life, and because the fat content of infant formula and follow-on formula is regulated (40–55 E% in infant formula and 35–55 E% in follow-on formula) (141), no further recommendations are given for the first 4–6 months of life. Because of the rapid growth rate during infancy, fat accounts for about 50% of the total energy intake in human milk and infant formula.

Regarding children 6–23 months, after 6 months of age, this high energy density provided by human milk is reduced with increasing amounts of foods. Thus, the fat intake can decline rapidly to around 30 E% at the end of infancy depending on the composition of the complementary food and the extent of partial breastfeeding. If the proportion of fat and, therefore, the energy density of the diet becomes too low in the first year or in early childhood, this might result in insufficient energy intake because children of this age have limited capability for ingesting more voluminous servings.

According to the EFSA, total fat intake below 25 E% has been associated with low vitamin intakes in some young children (142). The US Institute of Medicine has set an adequate intake (AI) for 7–12 month olds to 40 E% (143). Similarly, the EFSA has set an AI at 40 E% for children aged 7–12 months based on AI and consensus reports (142, 144). In the NNR 2023, the intake of total fat for infants between 6 and 11 months of age is recommended to be kept between 30 and 45 E%, and this is the same as in the NNR2012.

The quality of dietary fat is also important in infancy and childhood. From the age of 12 months, the intake of SFA should be less than 10 E%. The intake of TFA both from dairy fat and partially hydrogenated, industrially produced fats should be kept as low as possible. Partial breastfeeding is recommended from 6 months and throughout the child’s first year and can be continued for as long as it suits the mother and the child. Half or more of the energy from human milk is fat. Typical fatty acid composition (wt%) in mature breastmilk is 40–45% SFA, 40–45% MUFA, and 13–16% PUFA.

For children 6–23 months of age, the diet should constitute of 5–10 E% PUFA including at least 1 E% from n-3 fatty acids, including DHA, meaning that the recommendation is similar as for adults.

### Data gaps for future research

More research is needed in order to understand the protective associations between ruminant TFA and odd-chain fatty acids and risk of T2D and CVD found in observational studies. More research is needed to address the potential impact of dietary fat type on musculoskeletal and mental health. More research is needed to investigate the potential food source-specific effects of SFA. Interactions between nutrients, other compounds, and the physical structure of a food may influence the effect of SFA on risk markers, so-called food-matrix effects. This has been clearly demonstrated for dairy products (145, 146). However, although some types of dairy products may not exert as negative health effects as would be expected due to their content of SFA, both short-term randomized trials and observational cohort studies favor sources of unsaturated fat in direct comparisons (147, 148). More research is needed to understand the role of fermented full-fat dairy products such as cheese and yogurt in healthy dietary patterns.

### Limitations

Evidence was identified in the scientific literature only since 2011 and the search was performed in one database. Therefore, there might be some relevant older studies which did not meet inclusion criteria. Furthermore, as the search only included SRs and meta-analyses, there may be individual studies of high quality (not included in any SR) that have not been considered. Most of the evidence on disease outcomes is based on observational data, and although statistical adjustment for potential confounders is typically performed, residual confounding likely remains. Data on dietary intake are most often self-reported and various degrees of misreporting, and thus misclassification, can be expected. Although qualified SRs were available for many health outcomes, they were not available for all health outcomes. For cancer, few qualified SRs were available. For multiple individual cancer types, the few SRs available generally had low quality. For other health outcomes, such as osteoporosis, mental health, and muscle mass and function, the overall evidence is limited, and more high-quality research is needed before any conclusions can be drawn. An overall limitation when studying the association between fatty acid classes and health outcomes is that potential modifying effects by food source are lost, potentially contributing to heterogeneity. This issue has been of special interest in relation to dietary SFA, where observational studies suggest differential associations depending on the food source. Another general limitation that can be discussed is the statistical modeling of the dietary data, where specific substitution models are generally not available for many outcomes, which may hamper the overall interpretation and advice on practical implementation.
